# Combination of midostaurin and ATRA exerts dose-dependent dual effects on acute myeloid leukemia cells with wild type FLT3

**DOI:** 10.1186/s12885-022-09828-2

**Published:** 2022-07-09

**Authors:** Hao Lu, Xiang-qin Weng, Yan Sheng, Jing Wu, Hui-min Xi, Xun Cai

**Affiliations:** Shanghai Institute of Hematology, State Key Laboratory of Medical Genomics, National Research Center for Translational Medicine at Shanghai, Ruijin Hospital, Shanghai Jiao Tong University School of Medicine, No.197 Rui-jin Road II, Shanghai, 200025 China

**Keywords:** Acute myeloid leukemia, All-trans retinoic acid, Apoptosis, Differentiation, Midostaurin

## Abstract

**Background:**

Midostaurin combined with chemotherapy is currently used to treat newly diagnosed acute myeloid leukemia (AML) patients with FMS-like tyrosine kinase 3 (FLT3)-mutations. However, midostaurin acts as an antagonist to some chemotherapeutic agents in leukemia cell lines without FLT3 mutations. All-trans retinoic acid (ATRA) induces apoptosis when used in combination with midostaurin in FLT3-mutated AML cells. This combination has been shown to be safe in AML patients. However, the effect of this combination has not been investigated in AML without FLT3 mutations.

**Methods:**

Cell proliferation was assessed by a cell counting assay. Cell death was evaluated by cell viability and Annexin-V assays. Cell differentiation was assessed by CD11b expression profiling and morphological analysis. To explore the underlying mechanisms, we studied the role of caspase3/7, Lyn, Fgr, Hck, RAF, MEK, ERK, AKT, PU.1, CCAAT/enhancer binding protein β (C/EBPβ) and C/EBPε by Western blot analysis and immunoprecipitation assays. Antitumor activity was also confirmed in mouse xenograft models established with AML cells.

**Results:**

In this study, 0.1 − 0.25 μM midostaurin (mido(L)) combined with ATRA induced differentiation while 0.25 − 0.5 μM midostaurin (mido(H)) combined with ATRA triggered apoptosis in some AML cell lines without FLT3-mutations. Midostaurin combined with ATRA (mido-ATRA) also exhibited antitumor activity in mouse xenograft models established with AML cells. Mechanistically, mido(H)-ATRA-induced apoptosis was dependent on caspase-3/7. Mido(L)-ATRA inhibited Akt activation which was associated with decreased activity of Lyn/Fgr/Hck, resulted in dephosphorylation of RAF S259, activated RAF/MEK/ERK, along with upregulating the protein levels of C/EBPβ, C/EBPε and PU.1. A MEK specific inhibitor was observed to suppress mido(L)-ATRA-induced increases in the protein levels of C/EBPs and PU.1 and mido(L)-ATRA-induced differentiation. Furthermore, inhibition of Akt activity promoted mido(L)-ATRA-induced downregulation of RAF S259 phosphorylation and mido(L)-ATRA-induced differentiation. Therefore, Lyn/Fgr/Hck-associated Akt inhibition activated RAF/MEK/ERK and controlled mido(L)-ATRA-induced differentiation by upregulation of C/EBPs and PU.1. Mido(L)-ATRA also promoted assembly of the signalosome, which may facilitate RAF activation.

**Conclusions:**

Midostaurin combined with ATRA exerts antitumor activity against AML with wild-type FLT3 mutations in vitro and in vivo. These findings may provide novel therapeutic strategies for some AML patients without FLT3 mutations and imply a new target of midostaurin.

**Supplementary Information:**

The online version contains supplementary material available at 10.1186/s12885-022-09828-2.

## Background

Midostaurin was originally developed as an inhibitor of protein kinase C (PKC) but was later discovered to have potent inhibitory activities against platelet-derived growth factor receptors (PDGFRs), c-KIT, cyclin-dependent kinase 1 (CDK1), vascular endothelial growth factor (VEGF) and FMS-like tyrosine kinase 3 (FLT3) [1]. FLT3 belongs to the receptor tyrosine kinase family and is widely expressed on hematopoietic stem and progenitor cells [1]. FLT3 is mutated in approximately 30% of patients with acute myeloid leukemia (AML), and is the most prevalent molecular aberrancy in AML [2]. In 2017, midostaurin was approved by both the European Medicines Agency (EMA) and the United States Food and Drug Administration (FDA) for addition to intensive chemotherapy in patients with newly diagnosed FLT3-mutated AML [1].

Although midostaurin in combination with chemotherapy is currently in routine clinical use in patients with newly diagnosed FLT3-mutated AML, midostaurin has been identified as an antagonist to some chemotherapeutic agents, such as cytarabine, doxorubicin, idarubicin, mitoxantrone, etoposide, and vincristine in leukemia cell lines without FLT3 mutations [3–5]. Thus, midostaurin in combination with above chemotherapeutic agents may not be suitable for AML patients with wild-type (wt) FLT3. Midostaurin has shown synergistic effects with both 5-azacytidine and decitabine in wtFLT3 cell lines [3]. However, there are no strong clinical data to support the benefit of combinations of hypomethylating agents with FLT3 inhibitors in FLT3-mutated AML patients [6], and no clinical trial of these combinations has been carried out in AML patients with wtFLT3. Therefore, the development of other agents for use in combination with midostaurin to treat AML patients, especially patients without FLT3 mutations, is still needed.

The majority of AML patients are elderly patients who not tolerate intensive treatment and are unsuitable for stem cell transplantation [2]. Differentiation therapy, with comparatively less severe side effects, may be an alternative to chemotherapy for these elderly patients. Although the successful treatment of acute promyelocytic leukemia (APL) patients with all-trans retinoic acid (ATRA) is regarded as a milestone in tumor differentiation therapy, a clinical trial of ATRA in patients with non-APL AML had disappointing results [7, 8]. Since ATRA is a master regulator of myeloid cell differentiation, discovering strategies to sensitize AML cells to ATRA may lead to the development of ATRA-based treatments for patients with non-APL AML. The combination of ATRA and midostaurin has shown additive or synergistic effects in inducing apoptosis in FLT3 mutated AML cell lines [9]. Moreover, no dose-limiting toxicities were observed in a phase I study of the cladribine, cytarabine, granulocyte colony stimulating factor (CLAG) regimen, midostaurin and ATRA in patients with relapsed/refractory AML [10]. It is suggested that the combination of midostaurin and ATRA may be safe in AML patients. However, the effect of this combination has not been investigated in AML cell lines without FLT3 alterations.

In the present study, the wtFLT3 AML cell lines, HL-60 and U937 and the ATRA-resistant cell line HL-60Res [11] were used as in vitro models. A clinically achievable concentration of midostaurin was used. In these three cell lines, high-dose (0.25 − 0.5 μM) midostaurin (mido(H)) combined with ATRA triggered apoptosis, while low-dose (0.1 − 0.25 μM) midostaurin (mido(L)) enhanced ATRA-induced differentiation. The combination of midostaurin and ATRA (mido-ATRA) exhibited antitumor activity in mouse xenograft models established with AML cells. Mechanistically, mido(H)-ATRA-induced apoptosis was dependent on caspase-3/7. Lyn/Fgr/Hck-associated Akt inhibition activated RAF/MEK/ERK and controlled mido(L)-ATRA-induced differentiation by upregulating CCAAT/enhancer binding proteins (C/EBPs) and PU.1. Signalosome assembly promoted by mido(L)-ATRA may also facilitate RAF activation.

## Methods

### Reagents

ATRA was obtained from Sigma-Aldrich (St Louis, MO, USA). Midostaurin**,** LY294002, and U0126 were purchased from Selleckchem Chemicals (Houston, TX, USA). All reagents were dissolved in dimethyl sulfoxide (DMSO, Sigma-Aldrich) for in vitro studies. For in vivo experiments, midostaurin and ATRA were dissolved in Cremophor EL (MedChemExpress, Princeton, NJ, USA):ethanol (50:50) at 50 mg/mL and 10 mg/mL, respectively, and stored at 4 °C in the dark. These two stock solutions were diluted fourfold with water on the day of use.

### Cell culture and cell viability

HL-60 and HL-60Res cells were cultured in Iscove’s modified Dulbecco’s medium(IMDM)(GE healthcare Biosciences, Uppsala, Sweden), supplemented with 20% fetal bovine serum (GE healthcare Biosciences). U937 cells were cultured in RPMI-1640 medium (GE healthcare Biosciences) supplemented with 10% fetal bovine serum in a humidified atmosphere of 95% air and 5% CO_2_ at 37 ºC. Cell viability was assessed by trypan blue exclusion as previously described [12].

### Cell differentiation assays

Cell differentiation was assessed by cellular morphology and the content of cells expressing the cell surface differentiation-related antigen CD11b as previously described [12]. Morphology was evaluated with May-Grunwald-Giemsa staining and observed at 1000 × magnification. Flow cytometry (EPICS XL, Coulter, Hialeah, FL, USA) was used to analyze the expression of the cell surface differentiation-related antigen CD11b (Coulter, Marseilles, France).

### Annexin-V assay

Annexin-V was analyzed with an Annexin V-7AAD Apoptosis Detection Kit (BD Biosciences Pharmingen, San Diego, CA, USA) according to the manufacturer’s instructions. Briefly, 5 × 10^5^ cells were collected and washed with binding buffer. Then, the cells were incubated with 5 μL of 7-amino-actinomycin and 5 μL of annexin-V at room temperature in the dark for 15 min. Fluorescence intensities were assessed by flow cytometry.

### Western blot analysis

Protein lysate preparation and immunoblotting were carried out as previously described [13]. Briefly, after lysis with RIPA buffer (Beyotime Biotechnology Ltd., Shanghai, China) and centrifugation at 13,000 rpm at 4 ºC for 10 min, the supernatants were harvested, and proteins were quantified by a protein quantification kit (Beyotime Biotechnology Ltd.). Protein extracts (50 or 20 μg) were loaded onto 8% SDS polyacrylamide gels and subjected to electrophoresis, and transferred to polyvinylidene difluoride membranes (GE Healthcare UK Ltd, Buckinghamshire, UK). Most of the membranes were cut into the appropriate sizes and blocked with 5% bovine albumin or 5% nonfat milk in phosphate-buffered saline (PBS), incubating with the following primary antibodies: anti-phospho-p44/42 Erk1/2 (Thr202/Tyr204), Erk1/2, phospho-MEK1/2 (Ser217/221),MEK1/2, phospho-Akt (Ser473), phospho-Akt (Thr308), AKT, p-RAF-1(Ser259), p-RAF-1(Ser338), RAF-1 and Phospho-Src family (Tyr416) (all from Cell Signaling Technology, Beverly, MA, USA); Fgr, Lyn, Hck, C/EBPε, C/EBPβ, PU.1, p-RAF-1(Tyr340/341), caspase-7 and caspase-3 (all from Santa Cruz Biotech, Santa Cruz, CA); GAPDH (Proteintech, Rosemont, IL, USA) and p-cRAF (Ser621) (Invitrogen Corporation, Camarillo, CA, USA). Then, the membranes were incubated with a horseradish peroxidase (HRP)-conjugated secondary antibody (GE Healthcare UK Ltd). Immunocomplexes were visualized by a chemiluminescence kit (GE Healthcare UK Ltd.) according to the manufacturer’s instructions. The densities of protein bands were quantified using ImageJ software (National Institutes of Health, Bethesda, MD) and were expressed as the level of the target protein relative to that of GAPDH or the immunoprecipitated bait protein, and defined as 1.0 for DMSO-treated cells.

### Immunoprecipitations

Immunoprecipitation was carried out as previously described [13]. Briefly, after lysis in RIPA buffer and centrifugation at 13,000 rpm at 4 °C for 10 min, the lysates were incubated separately with 2 μg of the anti-lyn, anti-Fgr, anti-Hck or anti-RAF antibody overnight at 4 °C. Then, the lysates were incubated with Protein G Plus/ Protein A-Agarose (Santa Cruz Biotech) for 2 h at 4 °C. After centrifugation and three washes with lysis buffer, immunocomplexes were collected and boiled in 2 × Laemmli reducing buffer for 10 min. The immunocomplexes were analyzed by immunoblotting. When the membrane was incubated with anti-lyn, anti-Fgr, anti-Hck or anti-Phospho-Src family (Tyr416) antibodies, mouse anti-rabbit IgG (light-chain specific) (Cell Signaling Technology) was used to avoid the interference with Ig G heavy chain.

### Tumor Xenograft Experiments

Five- to six-week-old female non obese diabetic/severe combined immunodeficient (NOD/SCID) mice were purchased from Beijing Vital River Laboratory Animal Technology Co., Ltd. (Beijing, China). Animal handling was authorized by the committee for humane treatment of animals at Shanghai Jiao Tong University School of Medicine and followed the National Research Council's Guide for the Care and Use of Laboratory Animals. Mice were injected subcutaneously with 5 × 10^6^ cells. When tumors ranging in size from 100 to 150 mm^3^ had been established in all mice, the mice were randomly assigned to receive midostaurin (50 mg/kg, p.o.) or/and ATRA (10 mg/kg, i.p.) daily. Body weights and tumor dimensions were recorded every two days. Tumor volume was calculated using the standard formula, volume (mm^3^) = π/6 × width^2^ × length. When the tumor volume in the vehicle group was approximately 2000 to 2500 mm^3^, treatment was terminated and mice were euthanized by carbon dioxide inhalation. Tumors were peeled and weighed.

### Statistical analysis

For analysis of cell viability, cell growth, tumor volume, tumor weight, Annexin-V staining and the content of CD11b^+^ cells, values are expressed as the means ± SDs, and p values are mentioned in the corresponding figure legends. The chi-square test (*n* = 20,000) was used to analyze the flow-cytometric data for CD11b and Annexin-V assays. Cell viability, cell growth, tumor volume and tumor weight data were analyzed with one-way ANOVA, and *P* < 0.05 is considered to indicate statistical significance.

## Results

### Low-dose midostaurin and ATRA induce differentiation and high-dose midostaurin and ATRA trigger apoptosis in AML cells without Flt3 mutations

**Fig. 1:**
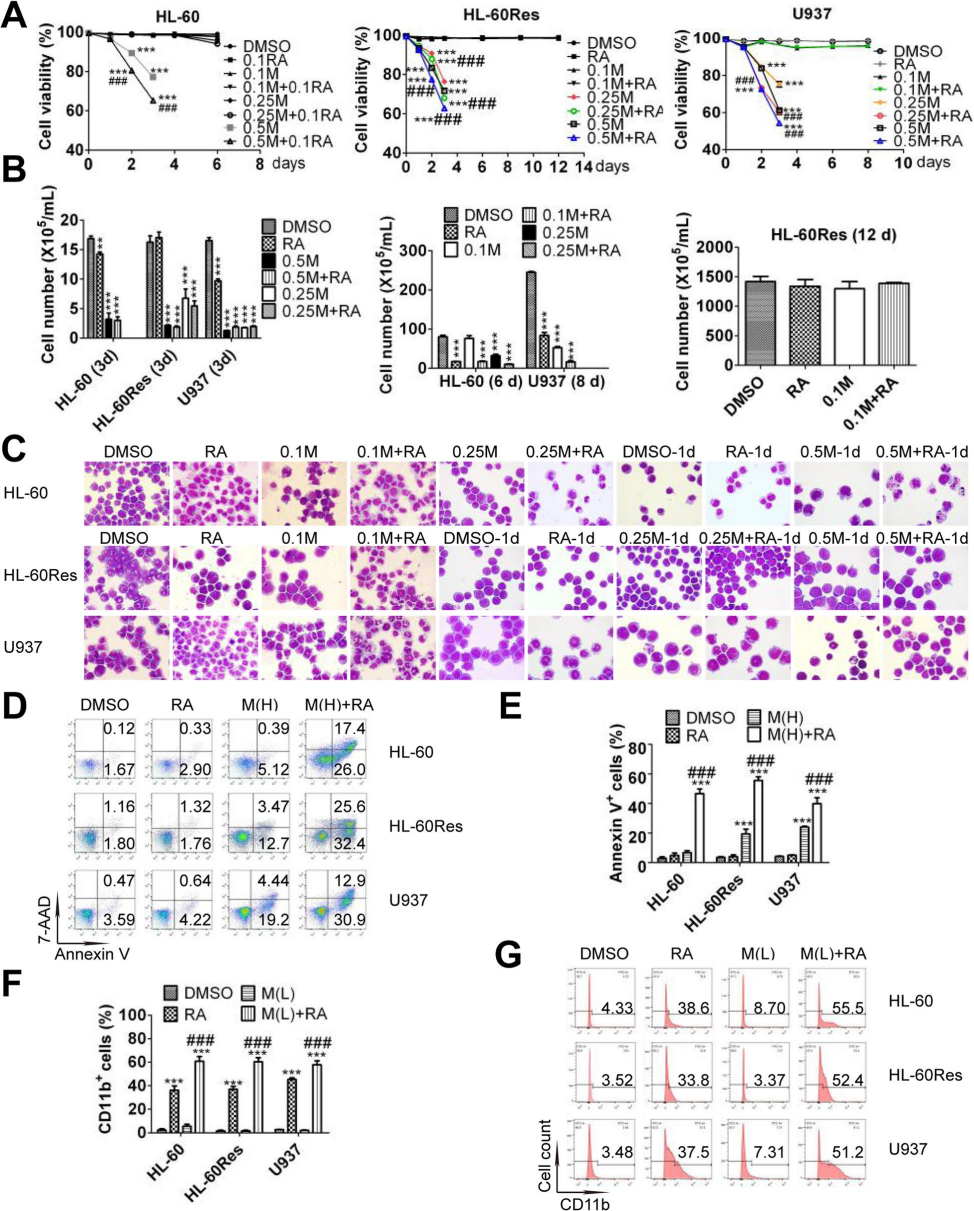
Mido(L)-ATRA induces differentiation and mido(H)-ATRA triggers apoptosis. (**A**) The cell viability upon the indicated treatments. Each value indicates the mean ± SD of triplicate samples. ***P < 0.005 versus DMSO-treated cells. ### *P* < 0.005 versus same concentrations of mido-treated cells. The results are representative of three independent experiments. (**B**) The cell growth of the three cell lines on the last day with the indicated treatment. **P < 0.01, ***P < 0.005 versus DMSO-treated cells. Each value indicates the mean ± SD of triplicate samples. The results are representative of three independent experiments. (**C**) HL-60 cells were treated with 0.1–0.25 μM midostaurin and/or 0.1 μM ATRA for 6 d or with 0.5 μM midostaurin and/or 0.1 μM ATRA for 1 d. HL-60Res and U937 cells were treated with 0.1 μM midostaurin and/or 1 μM ATRA for 12 and 8 d, respectively, or with 0.25–0.5 μM midostaurin and/or 1 μM ATRA for 1 d. Representative morphological images. Magnification is 1,000 × . (**D**) Cells were treated with 0.5 μM midostaurin (M(H)) and/or ATRA for 2 d. Scatter plots of Annexin V expression. The results are representative of three independent experiments. (**E**) The column graph of Annexin V expression in the three cell lines treated as described in (**D**). Each value indicates the mean ± SD of three independent measurements. ***P < 0.005 versus DMSO-treated cells. ### *P* < 0.005 versus mido (H)-treated cells. (**F**) HL-60 cells were treated with 0.25 μM midostaurin (M(L)) and/or 0.1 μM ATRA for 6 d. HL-60Res and U937 cells were treated with 0.1 μM midostaurin (M(L)) and/or 1 μM ATRA for 12 and 8 d, respectively. The column graph of CD11b expression. Each value indicates the mean ± SD of three independent measurements. ***P < 0.005 versus DMSO-treated cells. ### *P* < 0.005 versus ATRA-treated cells. (**G**) The histograms of CD11b expression in the three cell lines treated as described in (**F**). The results are representative of three independent experiments

The dose of midostaurin in a phase I study of the CLAG regimen combined with midostaurin and ATRA was identified to be 50 mg bid, resulting in a median plateau trough concentration of 467 ng/mL, which is equivalent to 0.81 μM [10, 14]. For feasibility in future clinical applications, a clinically achievable concentration of 0.5 μM was used as the maximum concentration of midostaurin in this study. Cell viability was markedly decreased in HL-60 cells treated with 0.5 μM midostaurin and in U937 and HL-60Res cells treated with 0.25 − 0.5 μM midostaurin. The combination of ATRA with the above concentrations of midostaurin further promoted the midostaurin-induced decrease in cell viability in all cell lines (Fig. 1A). Cell proliferation was inhibited in HL-60 cells subjected to any other treatment except 0.1 μM midostaurin. Any concentrations of midostaurin and/or ATRA suppressed proliferation in U937 cells, while only 0.25 − 0.5 μM midostaurin with/without ATRA inhibited proliferation in HL-60Res cells (Fig. 1B). Morphologically, cells with a decreased nuclear/cytoplasmic ratio were observed among U937 and HL-60 cells but not HL-60Res cells treated with ATRA. Cells with kidney-shaped nuclei were observed among HL-60 and HL-60Res cells cotreated with 0.1 μM midostaurin and ATRA. Fully differentiated cells with lobed nuclei were observed among HL-60 cells cotreated with 0.25 μM midostaurin and ATRA and among U937 cells cotreated with 0.1 μM midostaurin and ATRA. Some apoptotic cells were observed among HL-60 cells cotreated with 0.5 μM midostaurin and ATRA and among HL-60Res cells cotreated with 0.25 − 0.5 μM midostaurin and ATRA. More apoptotic cells were observed among U937 cells cotreated with 0.25 − 0.5 μM midostaurin and ATRA than among those treated with 0.25 − 0.5 μM midostaurin alone (Fig. 1C). Consistent with the observations regarding morphology and cell viability, in all cell lines, more Annexin-V^+^ cells were observed among cells treated with the combination of 0.5 μM midostaurin and ATRA than among those treated with 0.5 μM midostaurin alone (Fig. 1D and E). The content of Annexin V^+^ cells was hardly affected by cotreatment with 0.1/0.25 μM midostaurin and ATRA in all cell lines (Supplementary Fig. 1). In addition, 0.1/0.25 μM midostaurin significantly promoted the ATRA-induced increase in the content of CD11b^+^ cells in all cell lines, while 0.5 μM midostaurin had limited effect on it (Fig. 1F, G and Supplementary Fig. 2). Therefore, low-dose (0.1 − 0.25 μM) midostaurin enhanced ATRA-induced differentiation, while ATRA promoted high-dose (0.25 − 0.5 μM) midostaurin-triggered apoptosis. Taken together, these findings indicate that low-dose (0.1 − 0.25 μM) midostaurin and ATRA induced differentiation, while high-dose (0.25 − 0.5 μM) midostaurin and ATRA triggered apoptosis.

### Mido(L)-ATRA activates the RAF/MEK/ERK signaling pathway and increases the protein levels of C/EBPs and PU.1, while mido(H)-ATRA activates caspase-3/7

**Fig. 2:**
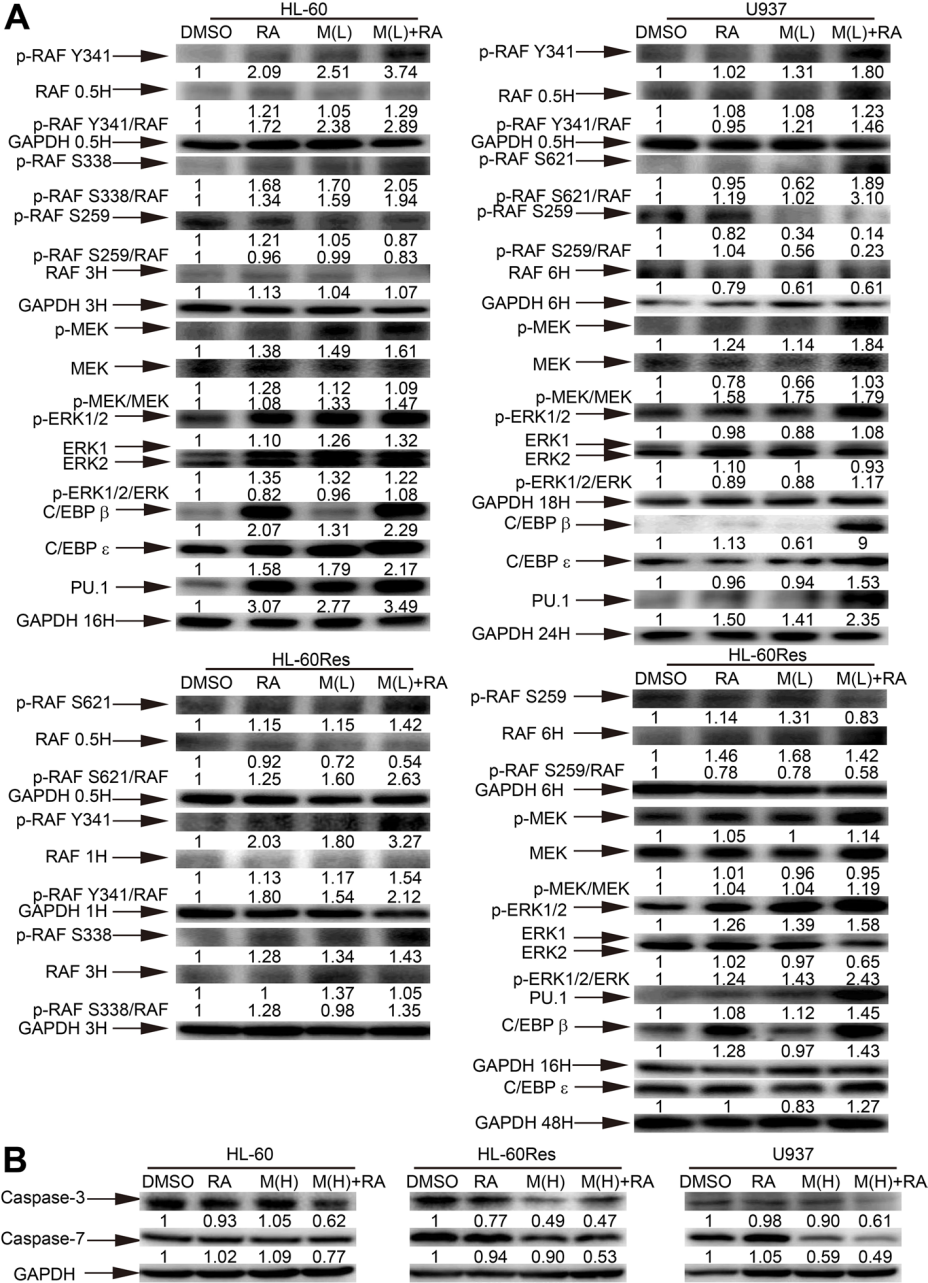
Mido(H)-ATRA activates caspase-3/7, and mido(L)-ATRA activates RAF-MEK-ERK and increases the expression of C/EBPs and PU.1. (**A**) HL-60 cells were treated with 0.25 μM midostaurin (M(L)) and/or 0.1 μM ATRA (RA). U937 and HL-60Res cells were treated with 0.1 μM midostaurin (M(L)) and/or 1 μM ATRA (RA). Changes in protein expression were detected at different time points and the corresponding expression of GAPDH at each time point was used as the internal control. (**B**) HL-60 cells were treated with 0.5 μM midostaurin (M(H)) and/or 0.1 μM ATRA (RA) for 3 h. U937 and HL-60Res cells were treated with 0.5 μM midostaurin (M(H)) and/or 1 μM ATRA (RA) for 12 and 24 h, respectively. GAPDH was used as the internal control. Most of the membranes were cut prior to hybridization and the original blots are presented in Supplementary Fig. 3. The values shown below each lane indicate relative units, with the values in DMSO-treated cells defined as 1.0. The phosphorylated protein/unphosphorylated protein ratios are also shown

Since low-dose midostaurin enhanced ATRA-induced differentiation in all cell lines and ATRA is a master regulator of myeloid cell differentiation, we focused on certain signaling pathways and proteins controlling ATRA-induced granulocytic differentiation. We first investigated the role of the RAF/MEK/ERK signaling pathway, which is required for ATRA-induced differentiation in the HL-60 cell line [15]. The phosphorylation of different RAF activation sites in these cell lines, such as, Y341 and S338 in HL-60 cells, S621 and Y341 in U937 cells, and Y341, S621 and S338 in HL-60Res cells, was increased with mido(L)-ATRA treatment. In all cell lines, the phosphorylation of the inhibitory site S259 was decreased with mido(L)-ATRA treatment. Moreover, the phosphorylation of MEK and ERK was increased more significantly with mido(L)-ATRA treatment than with ATRA treatment in all cell lines (Fig. 2A). C/EBPβ, C/EBPε and PU.1 are crucial transcription factors for myeloid differentiation [16]. Moreover, some medications induce myeloid differentiation in AML cells via MEK/ERK-mediated increase in the protein levels of C/EBPβ, C/EBPε and PU.1 [12, 13, 17, 18]. Compared with ATRA treatment, mido(L)-ATRA treatment increased the protein levels of C/EBPβ, C/EBPε and PU.1 more markedly in all cell lines (Fig. 2A). Thus, mido(L)-ATRA activated RAF/MEK/ERK and increased the protein levels of C/EBPs and PU.1 in all cell lines.

The protein levels of caspase-3 and caspase-7 were decreased with mido(H)-ATRA treatment in all cell lines (Fig. 2B). Therefore, mido(H)-ATRA activated caspase3/7 in all cell lines.

### Mido(L)-ATRA induces differentiation via RAF/MEK/ERK-mediated modulation of the protein levels of C/EBPs and PU.1

**Fig. 3:**
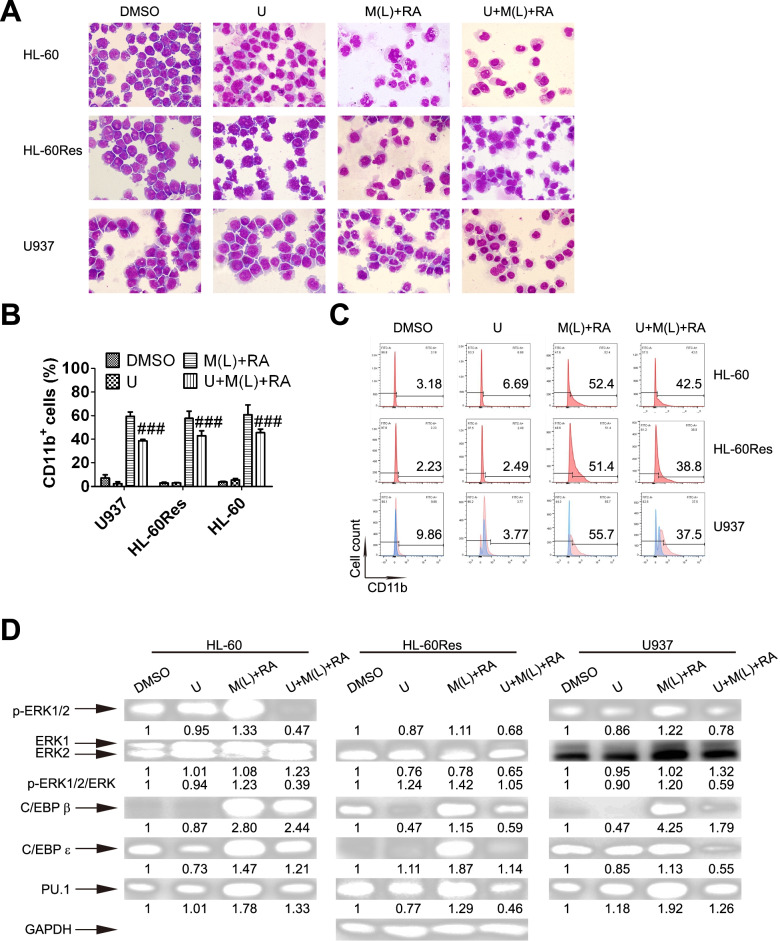
Mido(L)-ATRA induces differentiation via MEK-ERK-mediated modulation of the protein levels of C/EBPs and PU.1. HL-60, HL-60Res and U937 cells were pretreated with 1, 5 and 5 μM U0126 (U), respectively, for 2 h. Then, HL-60 cells were treated with 0.25 μM midostaurin (M(L)) and/or 0.1 μM ATRA (RA) for 4 d. U937 and HL-60Res cells were treated with 0.1 μM midostaurin (M(L)) and/or 1 μM ATRA (RA) for 4 and 8 d, respectively. (**A**) The effect of U0126 on the morphology of HL-60, U937 and HL-60Res cells treated with low-dose midostaurin and ATRA. The magnification is 1,000 × . The results are representative of three independent experiments. Differentiation was also assessed by flow cytometric analysis of CD11b expression in the three cell lines. (**B**) The column graph of flow-cytometric analysis of CD11b expression in the three cell lines. Each value represents the mean ± SD of three independent measurements. ### *P* < 0.001 compared with mido(L) + RA. (**C**) The histograms of flow cytometric analysis of CD11b expression in the three cell lines with the indicated treatment. U937 cells not labeled with the anti-CD11b antibody were used as negative controls, as shown in blue, while U937 cells labeled with the anti-CD11b antibody are shown in pink. The content of CD11b.^+^ cells was deducted from the autofluorescence contribution. The results are representative of three independent experiments. (**D**) Western blot analysis of phosphorylated ERK1/2, C/EBPβ, PU.1 and C/EBPε in the three cell lines with the indicated treatments for 16 h. The expression of GAPDH was used as the internal control. The membranes were cut prior to hybridization and the original blots are presented in Supplementary Fig. 3. The values shown below each lane represent relative units, with the values in DMSO-treated cells defined as 1.0. The phosphorylated ERK/ERK ratios are also shown

To further assess the role of RAF/MEK/ERK in mido(L)-ATRA-induced differentiation, cells were pretreated with U0126, a specific inhibitor of MEK. With U0126 pretreatment, the differentiated cells with lobed or kidney-shaped nuclei induced by mido(L)-ATRA treatment were replaced by primitive cells with round nuclei and a large nuclear/cytoplasmic ratio in all cell lines (Fig. 3A). Consistent with the morphology, the mido(L)-ATRA-induced increases in the content of CD11b^+^ cells were also suppressed by U0126 in all cell lines (Fig. 3B and C). It should be noted that since U0126 exhibits autofluorescence in U937 cells, U937 cells not labeled with the anti-CD11b antibody were used as negative controls, as shown in blue. The content of CD11b^+^ cells was deducted from the autofluorescence contribution (Fig. 3C). In addition, U0126 reduced the activity of MEK, as evaluated by the phosphorylation of its substrate ERK, in all cell lines. Moreover, U0126 also suppressed the mido(L)-ATRA-induced increases in the protein levels of C/EBPs and PU.1 in all cell lines (Fig. 3D). Therefore, mido(L)-ATRA induced differentiation via RAF/MEK/ERK-mediated modulation of the protein levels of C/EBPs and PU.1.

### Mido(L)-ATRA promotes the interactions between RAF and Lyn/Fgr/ Hck

**Fig. 4:**
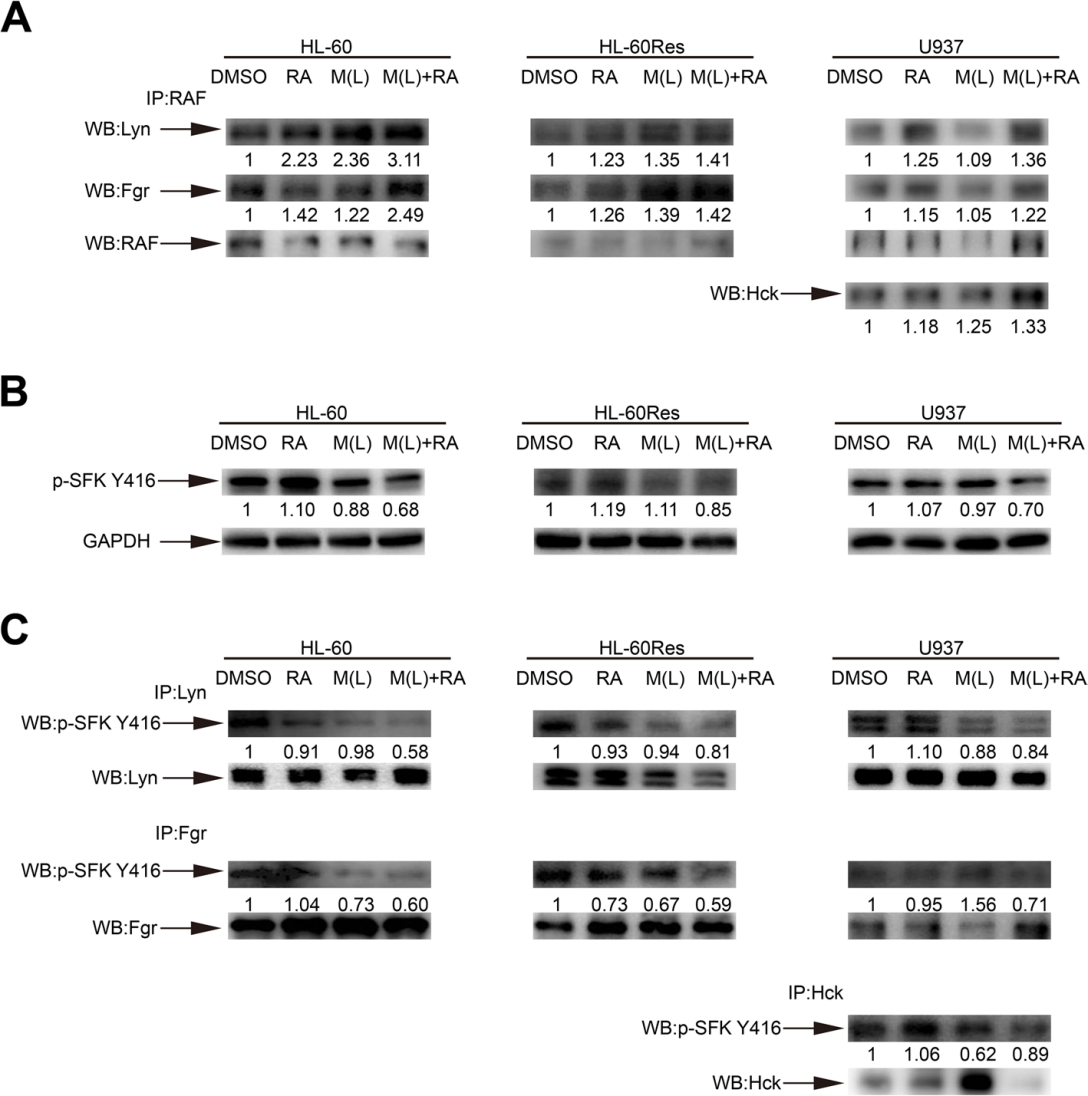
Mido(L)-ATRA promotes the interactions between RAF and Lyn/Fgr/Hck and inhibits the activities of Lyn/Fgr/Hck. HL-60, HL-60Res and U937 cells were treated as indicated for 2, 1 and 1 h, respectively. (A) RAF was immunoprecipitated, followed by Western blot analysis of Lyn, Fgr and Hck. (B) Phospho-Src Y416 was analyzed by Western blotting in whole-cell lysates of the three cell lines. GAPDH was used as the internal control. (C) Fgr and Lyn in HL-60 and HL-60Res cells and Lyn, Fgr and Hck in U937 cells were immunoprecipitated, followed by Western blot analysis of phospho-Src Y416. The same membrane incubated with the antibody against phospho-Src Y416 was stripped, and detection of Lyn, Fgr and Hck was then performed. The full length blots are presented in Supplementary Fig. 3. The values shown below each lane indicate relative units, with the values in DMSO-treated cells defined as 1.0

The Src family kinase (SFK) family is the largest subfamily of nonreceptor protein tyrosine kinases. Lyn and Fgr are the main SFKs in HL-60 cells, and Lyn, Fgr and Hck are the predominant SFKs in U937 cells [19, 20]. A macromolecular signaling complex, a signalosome, containing Lyn, Fgr, RAF/MEK/ERK, Vav1, SLP-76, c-Cbl, IRF-1 and CK2 is assembled to facilitate activation of the RAF/MEK/ERK signaling pathway in ATRA-induced differentiation of HL-60 cells [21]. RAF, Lyn and Fgr are the core components of the signalosome. To further explore the mechanisms of RAF activation, we first investigated whether mido(L)-ATRA promotes the interactions between RAF and SFKs. Total RAF was used as the bait protein for immunoprecipitation. Compared with ATRA, mido(L)-ATRA significantly promoted the interactions between RAF and Lyn/Fgr in HL-60 and HL-60Res cells and the interactions between RAF and Lyn/Fgr/Hck in U937 cells (Fig. 4A). Thus, mido(L)-ATRA enhanced signalosome assembly in all cell lines.

### Mido(L)-ATRA inhibits the activities of Lyn, Fgr and Hck

Although there is no evidence implicating SFKs as primary drivers of leukemia, several studies have shown high activity of Lyn in primary AML and indicated its crucial role in maintaining cell survival and proliferation of AML cells [22, 23]. Moreover, inhibition of SFKs enhances ATRA-induced differentiation in AML cells [24]. As an inhibitor of tyrosine kinases, midostaurin may inhibit the activity of SFK, leading to mido(L)-ATRA-induced differentiation. To explore the role of SFKs in mido(L)-ATRA-induced differentiation, we first investigated whether mido(L)-ATRA affects the activities of SFKs. Before dephosphorylation of RAF S259, the phosphorylation of SFK was decreased with mido(L)-ATRA treatment in all cell lines (Fig. 4B). To further identify the SFK whose activity was suppressed by mido(L)-ATRA treatment, Lyn and Fgr in HL-60 and HL-60Res cells and Lyn, Fgr and Hck in U937 cells were immunoprecipitated and then subjected to immunoblotting with an antibody against phospho-Src Y416. The phosphorylation of Lyn and Fgr in HL-60 and HL-60Res cells and the phosphorylation of Lyn, Fgr and Hck in U937 cells was suppressed with mido(L)-ATRA treatment (Fig. 4C). Thus, mido(L)-ATRA inhibited the activities of Lyn and Fgr in HL-60 and HL-60Res cells and the activities of Lyn, Fgr and Hck in U937 cells.

### Mido(L)-ATRA suppresses AKT activity to dephosphorylate RAF S259 and induce cell differentiation, and this mechanism is associated with SFK inhibition

**Fig. 5:**
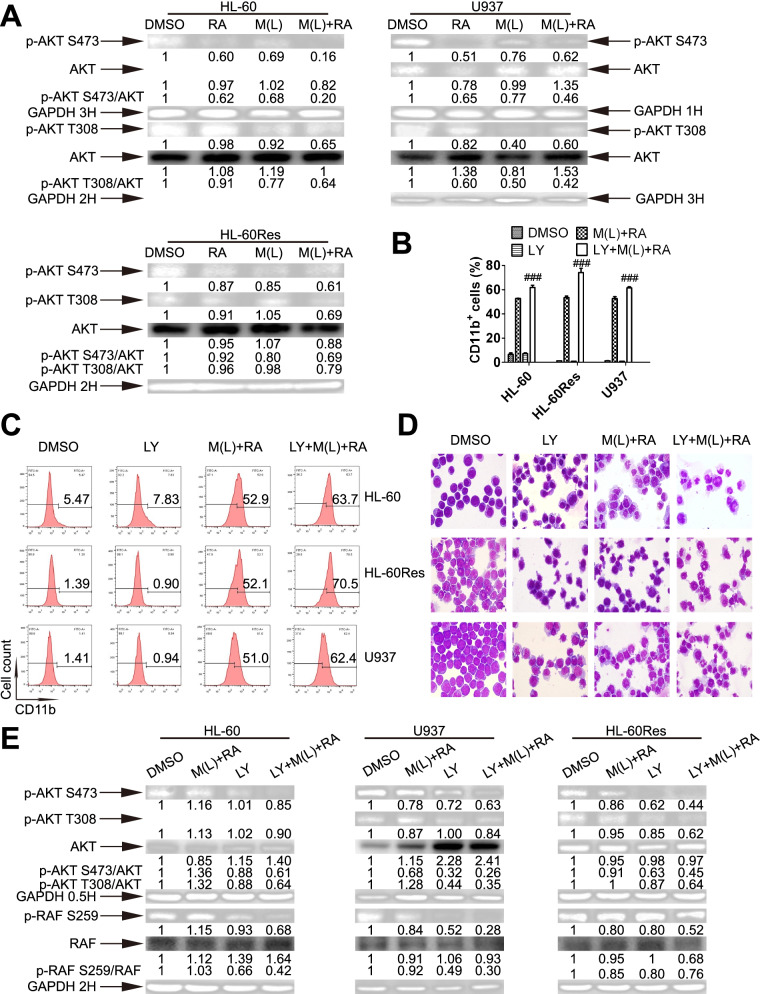
Mido(L)-ATRA suppresses AKT activity to dephosphorylate RAF S259 and induce cell differentiation. (**A**) Western blot analysis of phosphorylated Akt in the three cell lines. Changes in phosphorylation were detected at different time points, and the corresponding expression of GAPDH at each time point was used as the internal control. The membranes were cut prior to hybridization and the original blots are presented in Supplementary Fig. 3. The values shown below each lane indicate relative units, with the values in DMSO-treated cells defined as 1.0. The phosphorylated protein/unphosphorylated protein ratios are also shown. HL-60, HL-60Res and U937 cells were pretreated with 2, 2 and 0.5 μM LY294002 (LY) respectively, for 2 h. Then, HL-60 cells were treated with 0.25 μM midostaurin and 0.1 μM ATRA (M (L) + RA) for 3 d. U937 and HL-60Res cells were treated with 0.1 μM midostaurin and 1 μM ATRA (M (L) + RA) for 6 d. The effects of LY294002 on mido(L)-ATRA-induced differentiation were assessed by flow-cytometric analysis of CD11b expression (**B** and **C**) and the morphology (**D**, magnification is 1,000 × .) in the three cell lines. B shows the column graph of flow cytometric data of CD11b expression. ###*P* < 0.005 versus M(L) + RA-treated cells. Each value indicates the mean ± SD of three independent measurements. (**C**) The histograms of flow cytometric data of CD11b expression. The results are representative of three independent experiments. (**E**) HL-60, HL-60Res and U937 cells were pretreated with LY294002 (LY), and were then treated with M(L) and ATRA as mentioned above for 0.5 and 2 h. Phosphorylation of Akt and RAF S259 was analyzed by Western blotting in the three cell lines. GAPDH was used as the internal control. The membranes were cut prior to hybridization and the original blots are presented in Supplementary Fig. 3. The values shown below each lane indicate relative units, with the values in DMSO-treated cells defined as 1.0. The phosphorylated protein/unphosphorylated protein ratios are also shown

Lyn, Fgr or Hck is correlated with the activation of AKT, which can phosphorylate RAF S259 [15, 25–27]. Since mido(L)-ATRA inhibited the activities of Lyn, Fgr and Hck before dephosporylation of RAF S259 in all cell lines, we investigated whether dephosporylation of S259 was resulted from inactivation of Akt via Lyn/Fgr/Hck inhibition. Before RAF S259 dephosporylation, the phosphorylation of S473 and T308 of Akt was decreased in all cell lines treated with mido(L)-ATRA compared with ATRA (Fig. 5A). Moreover, in all cell lines, mido(L)-ATRA-induced differentiation was enhanced by LY294002, the specific inhibitor of the Akt upstream kinase PI3K, as determined by the content of CD11b^+^ cells and the morphology (Fig. 5B and D). Moreover, the phosphorylation of Akt S473, Akt T308 and RAF S259 was decreased in cells cotreated with LY294002 and mido(L)-ATRA (Fig. 5E). Thus, inhibition of Akt activity promoted mido(L)-ATRA-induced dephosphorylation of RAF S259. Therefore, mido(L)-ATRA suppressed the activity of Akt in association with SFK inhibition. Inhibition of Akt resulted in RAF S259 dephosphorylation, RAF activation and cell differentiation.

### In vivo efficacy of mido-ATRA against AML xenografts

**Fig. 6:**
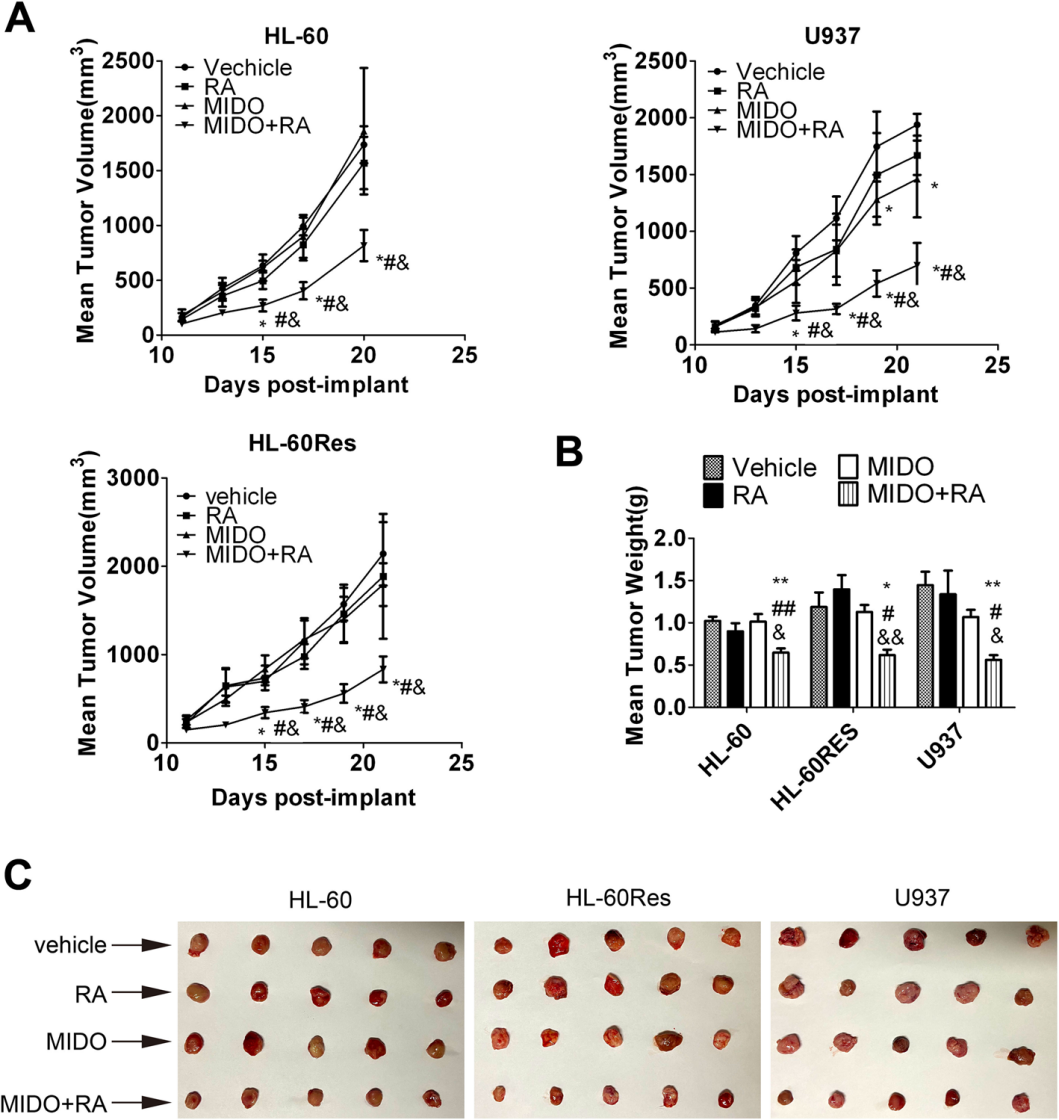
Mido-ATRA results in significant tumor regression in mouse xenograft models of AML. (**A**) Mean tumor volumes in mice injected with HL-60, HL-60Res and U937 cells and treated as indicated. *P < 0.05 versus vehicle-treated mice. # *P* < 0.05 versus mido-treated mice. & *P* < 0.05 versus ATRA-treated mice. (**B**) Weights of HL-60, HL-60Res and U937 cell xenografts harvested on Day 21. *P < 0.05, **P < 0.01 versus vehicle-treated mice. & *P* < 0.05, && *P* < 0.01 versus ATRA-treated mice. #*P* < 0.05, ##*P* < 0.01 versus mido-treated mice. (**C**) Images of HL-60, HL-60Res and U937 cell xenografts harvested on Day 21

To assess the in vivo antitumor activity of mido-ATRA, HL-60, HL-60Res and U937 subcutaneous tumor xenograft models were established in NOD/SCID mice. Mice bearing tumors with a volume of 100 to 150 mm^3^ were treated daily with ATRA (10 mg/kg, i.p.) or/and midostaurin (50 mg/kg, p.o.) for 10 consecutive days. No significant body weight loss or mortality was observed. ATRA did not result in tumor regression in any xenograft model while midostaurin had a modest effect on tumor regression only in U937 xenografts. However, the antitumor properties of mido-ATRA became apparent in all tumor xenograft models on Day 15 after implantation and were sustained over the course of the study (Fig. 6A). In addition, all tumor weights and sizes were significantly decreased with mido-ATRA, but tumor regression was not observed with midostaurin or ATRA monotherapy (Fig. 6B and C). Therefore, at the tolerable dose, mido-ATRA had significant antitumor effects in HL-60, HL-60Res and U937 xenograft models.

## Discussion

In the present study, the combination of a clinically achievable concentration of midostaurin and ATRA induced differentiation and apoptosis in some AML cells with wtFLT3 in a dose-dependent manner. Moreover, antitumor activity was demonstrated in xenografted mice treated with mido-ATRA. The safety of the combination of midostaurin and ATRA has been demonstrated in the past [10], suggesting that this combined therapy may have possible clinical applications. Therefore, the efficacy of mido-ATRA on primary cells from AML patients without FLT3 mutations merits further investigation.

Our study showed that mido(H)-ATRA activated caspase-3/7, thus, mido (H)-ATRA-induced apoptosis was caspase-3/7 dependent. Mido(L)-ATRA activated RAF/MEK/ERK and increased the protein levels of C/EBPs and PU.1 in all cell lines. Pretreatment with a specific inhibitor of MEK suppressed mido(L)-ATRA-induced differentiation and restored the protein levels of C/EBPs and PU.1 in these cell lines. Therefore, mido(L)-ATRA induced differentiation in these cell lines by RAF/MEK/ERK-mediated modulation of the protein levels of C/EBPs and PU.1. MEK and ERK have been reported to promote the expression of C/EBPβ and regulate the activities of PU.1 and C/EBPβ [28–30]. In addition, C/EBPβ activates the PU.1 promoter and PU.1 directly activates the transcription of C/EBPε [31, 32]. Thus, mido(L)-ATRA might induce differentiation via RAF-MEK-ERK- C/EBPβ-PU.1-C/EBPε cascade. Consistent with our results, this pathway is also involved in myeloid differentiation induced by other medications in AML cells [12, 13, 17, 18].

To investigate the mechanisms of RAF activation by mido(L)-ATRA, the signalosome and the initiation of RAF activation were taken into consideration. RAF, Lyn and Fgr constitute the ‘backbone’ of the signalosome to connect with other components of the signalosome [21]. In the present study, more Lyn and Fgr were present in the signalosome in cells treated with mido(L)-ATRA in all cell lines. Fgr has been confirmed to play a central role in signalosome activation by binding to NUMB, a scaffolding molecule in the signalosome [33]. Thus, the presence of Lyn and Fgr in the signalosome may facilitate the activation of RAF/MEK/ERK. In U937 cells, in addition to Fgr and Lyn, the content of Hck in the signalosome was increased with mido(L)-ATRA treatment. The investigation of Hck in the signalosome is limited, because all studies on the signalosome have been carried out only in HL-60 cells, in which Lyn and Fgr instead of Hck are the main SFKs. Due to the similarity of the catalytic domains of Hck and RAF and their upstream and downstream relationships [34, 35], there may be interactions between these proteins.

Initiation of RAF activation is mediated by dephosphorylation of RAF S259, on the other hand, S259 is phosphorylated by Akt, which is positively regulated by Lyn, Fgr or Hck [15, 25–27]. Mido(L)-ATRA inhibited the activities of Lyn and Fgr in HL-60 and HL-60Res cells, and the activities of Lyn, Fgr and Hck in U937 cells. After inhibition of SFK activity, AKT and RAF S259 were successively dephosphorylated in all cell lines treated with mido(L)-ATRA. Moreover, the Akt inhibitor promoted mido(L)-ATRA-induced inhibition of Akt activity, mido(L)-ATRA-induced dephosphorylation of RAF S259 and mido(L)-ATRA-induced differentiation in all cell lines. Therefore, inhibition of the activities of Lyn, Fgr and Hck by mido(L)-ATRA inactivated Akt, resulted in RAF S259 dephosphorylation, activated RAF/MEK/ERK, enhanced the protein levels of C/EBPs and PU.1 and finally induced myeloid differentiation. Similar to our results, Fgr inactivates RAF by phosphorylation of inhibitory sites in RAF and Lyn knockdown decreases the phosphorylation of RAF S259 [21, 36]. Inhibition of Lyn activity resulting in the suppression of Akt activity has also been observed in previous research [37]. Moreover, Lyn kinase activity is required for ATRA-induced phosphorylation of PI3K p85, the regulatory subunit of the upstream kinase of Akt [38].

The above results indicated that inhibition of SFK activity by mido(L)-ATRA promoted ATRA-induced differentiation in some AML cell lines without FLT3 mutations. Other tyrosine kinase inhibitors have also been shown to enhance ATRA-induced differentiation in AML cells by suppressing the activity of SFKs [24, 36, 37]. Thus, the kinase activity of SFKs may be the target of mido(L)-ATRA treatment. Whether the sensitivity of AML cells to mido(L)-ATRA is related to the activity of Lyn/Fgr/Hck needs to be further investigated. This research may be helpful for identifying AML patients suitable for mido(L)-ATRA treatment.

## Conclusions

Our present study demonstrated that a clinically achievable concentration of midostaurin combined with ATRA induced differentiation and apoptosis in AML cells without FLT3 mutations in a dose-dependent manner. Moreover, the antitumor activity of mido-ATRA was also observed in mouse xenograft models. Mechanistically, mido(H)-ATRA-induced apoptosis was dependent on caspase-3/7. Mido(L)-ATRA inhibited the activity of Lyn/Fgr/HCK, inactivated Akt, resulted in RAF S259 dephosphorylation, initiated RAF activation and activated downstream MEK/ERK signaling pathway, increased the protein levels of C/EBPs and PU.1 and finally induced myeloid differentiation. Signalosome assembly promoted by mido(L)-ATRA may also facilitate RAF activation. These findings may provide novel therapeutic strategies for some AML patients without FLT3 mutations and reveal the kinase activity of Lyn/Fgr/Hck as a new target of midostaurin. These results also imply that the kinase activity of Lyn/Fgr/Hck may be a therapeutic target in AML.

## Supplementary Information

**Additional file 1: Supplemental Figure 1.** The effect of Mido(L)-ATRA on the content of Annexin V+ cells. HL-60 cells were treated with 0.25 μM modistaurin (M(L)) and/or 0.1 μM ATRA for 6 d. HL-60Res and U937 cells were treated with 0.1 μM modistaurin (M(L)) and/or 1 μM ATRA for 12 and 8 d, respectively. (A) The column graph of the content of Annexin V+ cells in three cell lines. Each value represents the mean ± SD of three independent measurements. (B) Representative scattered plotgrams of Annexin V expression. Results were representative among three independent experiments. **Supplemental Figure 2.** The effect of Mido(H)-ATRA on the content of CD11b+ cells. Cells were treated with 0.5 μM midostaurin (M(H)) and/or ATRA for 2 d. (A) The column graph of CD11b expression in three cell lines. Each value represents the mean ± SD of three independent measurements. ***P<0.005, versus DMSO-treated cells. (B) Representative histograms of CD11b expression with high dose midostaurin and/or ATRA. Results were representative among three independent experiments. Supplemental Figure 3. Most membranes were cut prior to hybridization. Original blots of the immunoblot detection shown in Fig 2A-Fig 2B, Fig 3D, Fig 4A-Fig 4C, Fig 5A and Fig 5E.

## Data Availability

All data generated or analyzed during this study are included in this published article.

## Abbreviations

AML: Acute myeloid leukemia
APL: Acute promyelocytic leukemia
ATRA: All-trans retinoic acid
CDK1: Cyclin-dependent kinase 1
C/EBP: CCAAT/enhancer-binding protein
DMSO: Dimethyl sulfoxide
FLT3: FMS-related tyrosine kinase 3 receptor
KIT: Stem cell factor receptor
NOD/SCID: Female non obese diabetic/severe combined immunodeficient
PDGFR: Platelet-derived growth factor receptor
PKC: Protein kinase C
SFK: Src family kinase
VEGF: Vascular endothelial growth factor
