# Supplementary material for: Combination of midostaurin and ATRA exerts dose-dependent dual effects on acute myeloid leukemia cells with wild type FLT3
Source: BMC Cancer. 2022 Jul 9;22:749. doi: 10.1186/s12885-022-09828-2 (PMC9270826; doi:10.1186/s12885-022-09828-2)
Supplement: Supplementary file 1 — Additional file 1: Supplemental Figure 1. The effect of Mido(L)-ATRA on the content of Annexin V+ cells. HL-60 cells were treated with 0.25 μM modistaurin (M(L)) and/or 0.1 μM ATRA for 6 d. HL-60Res and U937 cells were treated with 0.1 μM modistaurin (M(L)) and/or 1 μM ATRA for 12 and 8 d, respectively. (A) The column graph of the content of Annexin V+ cells in three cell lines. Each value represents the mean ± SD of three independent measurements. (B) Representative scattered plotgrams of Annexin V expression. Results were representative among three independent experiments. Supplemental Figure 2. The effect of Mido(H)-ATRA on the content of CD11b+ cells. Cells were treated with 0.5 μM midostaurin (M(H)) and/or ATRA for 2 d. (A) The column graph of CD11b expression in three cell lines. Each value represents the mean ± SD of three independent measurements. ***P<0.005, versus DMSO-treated cells. (B) Representative histograms of CD11b expression with high dose midostaurin and/or ATRA. Results were representative among three independent experiments. Supplemental Figure 3. Most membranes were cut prior to hybridization. Original blots of the immunoblot detection shown in Fig 2A-Fig 2B, Fig 3D, Fig 4A-Fig 4C, Fig 5A and Fig 5E. [file 12885_2022_9828_MOESM1_ESM.pdf]

Supplementary information

Combination of midostaurin and ATRA exerts dose-dependent dual effects on acute myeloid leukemia cells with wild type *FLT3*

Hao Lu[1#], Xiang-qin Weng[1#], Yan Sheng[1], Jing Wu[1], Hui-min Xi[1], Xun Cai[1]*

1. Shanghai Institute of Hematology, State Key Laboratory of Medical Genomics, National Research Center for Translational Medicine at Shanghai, Ruijin Hospital, Shanghai Jiao Tong University School of Medicine, Shanghai, China

No.197 Rui-jin Road II, Shanghai 200025, China

***Corresponding author:**

Xun Cai

Shanghai Institute of Hematology, State Key Laboratory of Medical Genomics,

National Research Center for Translational Medicine at Shanghai, Ruijin Hospital,

Shanghai Jiao Tong University School of Medicine, Shanghai, China

No.197 Rui-jin Road II, Shanghai 200025, China

Tel: 86-21-64370045 extension 610709

Fax: 86-21-64743206

E-mail address: Cx10901@rjh.com.cn

#Hao Lu and Xiang-qin Weng contributed equally to this work.

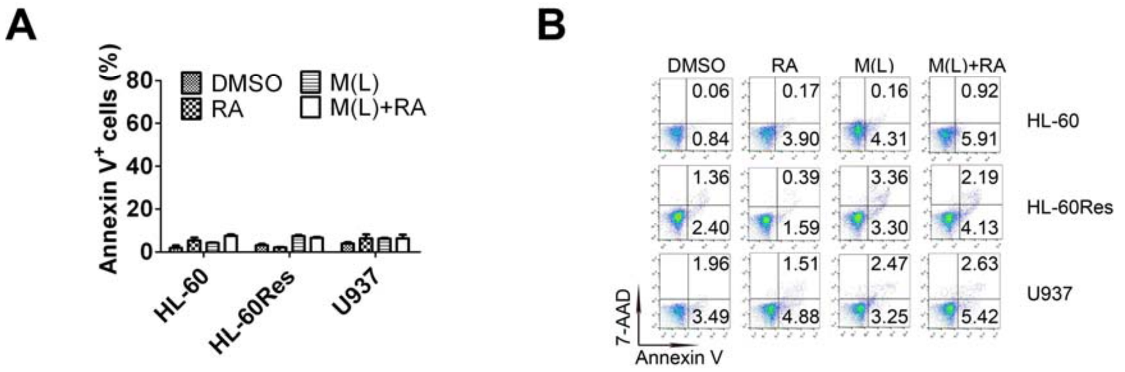

Supplemental Figure 1. The effect of Mido(L)-ATRA on the content of Annexin V^+ cells. HL-60 cells were treated with 0.25 μM modistaurin (M(L)) and/or 0.1 μM ATRA for 6 d. HL-60Res and U937 cells were treated with 0.1 μM modistaurin (M(L)) and/or 1 μM ATRA for 12 and 8 d, respectively. (A) The column graph of the content of Annexin V^+ cells in three cell lines. Each value represents the mean ± SD of three independent measurements. (B) Representative scattered plotgrams of Annexin V expression. Results were representative among three independent experiments.

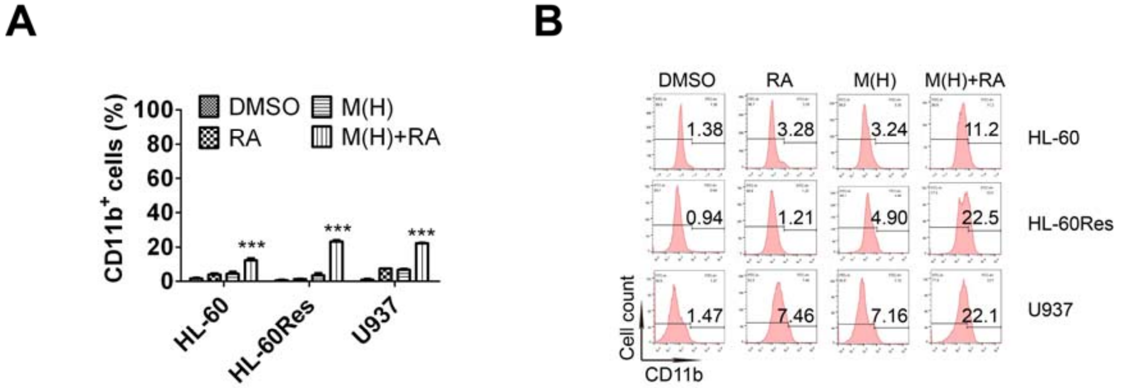

Supplemental Figure 2. The effect of Mido(H)-ATRA on the content of CD11b$^+$ cells. Cells were treated with 0.5 μM midostaurin (M(H)) and/or ATRA for 2 d. (A) The column graph of CD11b expression in three cell lines. Each value represents the mean ± SD of three independent measurements. ***P<0.005, versus DMSO-treated cells. (B) Representative histograms of CD11b expression with high dose midostaurin and/or ATRA. Results were representative among three independent experiments.

2A

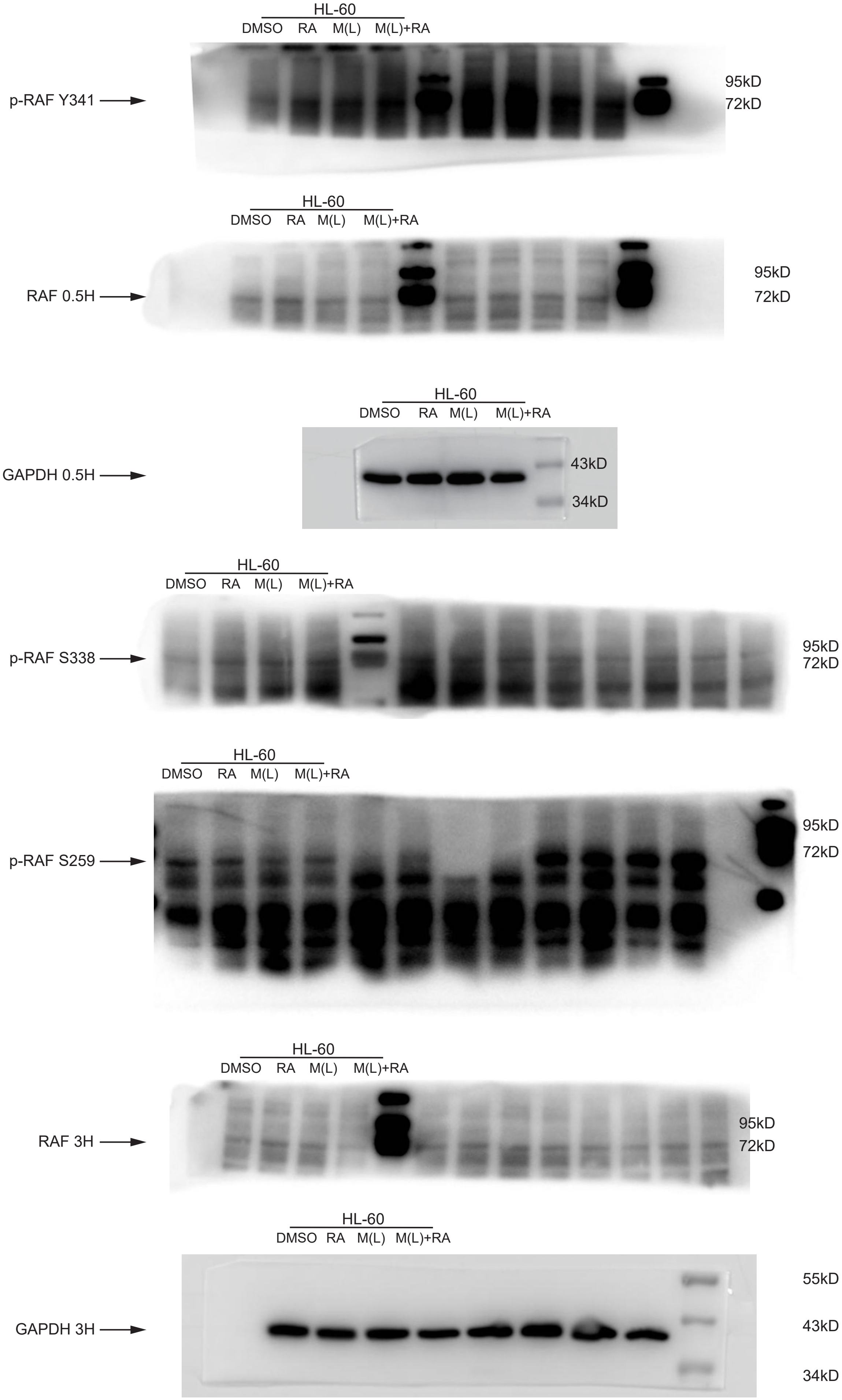

HL-60
DMSO RA M(L) M(L)+RA
p-RAF Y341
95kD
72kD
HL-60
DMSO RA M(L) M(L)+RA
RAF 0.5H
95kD
72kD
HL-60
DMSO RA M(L) M(L)+RA
GAPDH 0.5H
43kD
34kD
HL-60
DMSO RA M(L) M(L)+RA
p-RAF S338
95kD
72kD
HL-60
DMSO RA M(L) M(L)+RA
p-RAF S259
95kD
72kD
HL-60
DMSO RA M(L) M(L)+RA
RAF 3H
95kD
72kD
HL-60
DMSO RA M(L) M(L)+RA
GAPDH 3H
55kD
43kD
34kD

2A

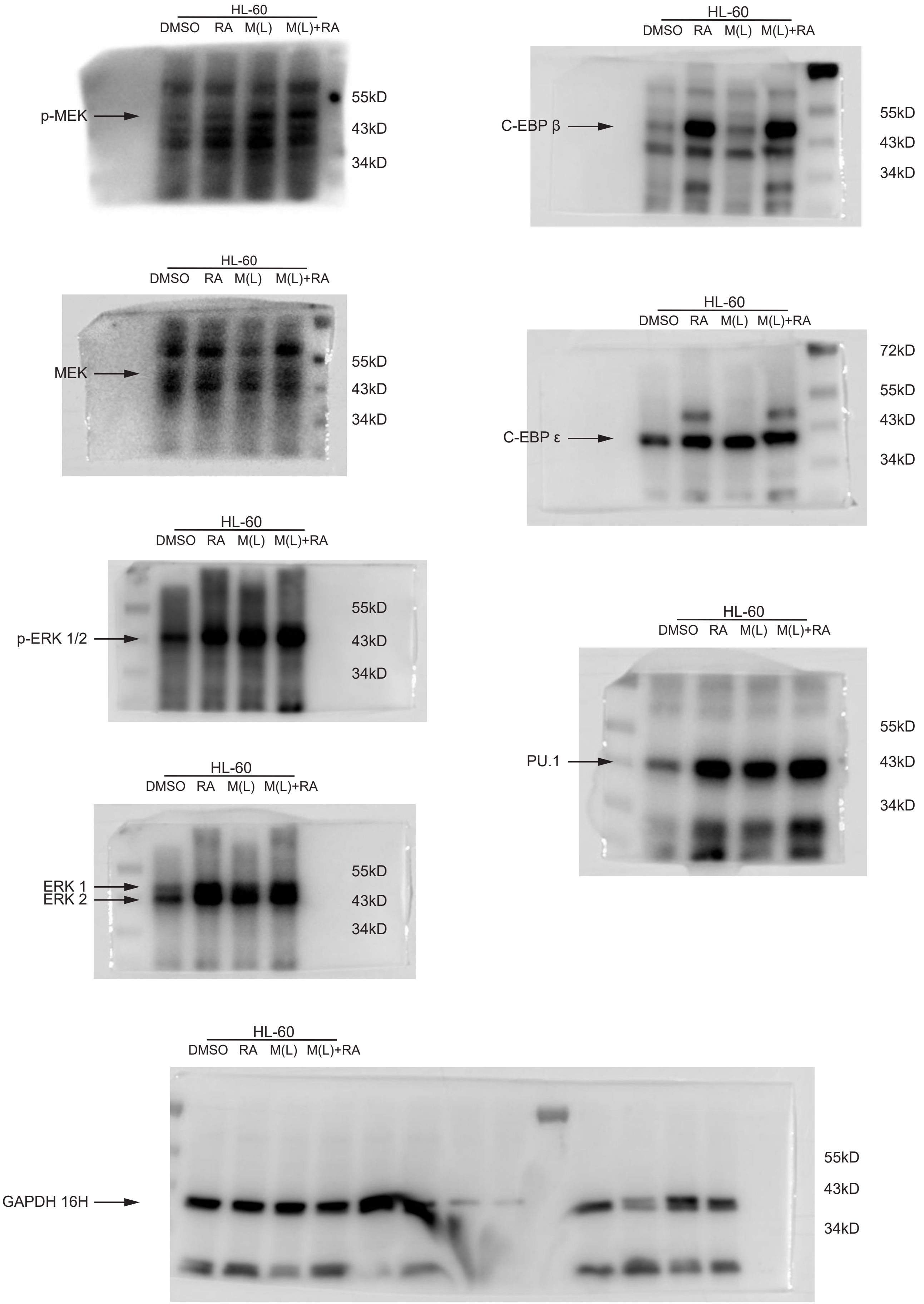
HL-60
DMSO RA M(L) M(L)+RA
p-MEK
55kD
43kD
34kD
HL-60
DMSO RA M(L) M(L)+RA
C-EBP β
55kD
43kD
34kD
HL-60
DMSO RA M(L) M(L)+RA
MEK
55kD
43kD
34kD
HL-60
DMSO RA M(L) M(L)+RA
C-EBP ε
72kD
55kD
43kD
34kD
HL-60
DMSO RA M(L) M(L)+RA
p-ERK 1/2
55kD
43kD
34kD
HL-60
DMSO RA M(L) M(L)+RA
PU.1
55kD
43kD
34kD
HL-60
DMSO RA M(L) M(L)+RA
ERK 1
ERK 2
55kD
43kD
34kD
HL-60
DMSO RA M(L) M(L)+RA
GAPDH 16H
55kD
43kD
34kD

2A

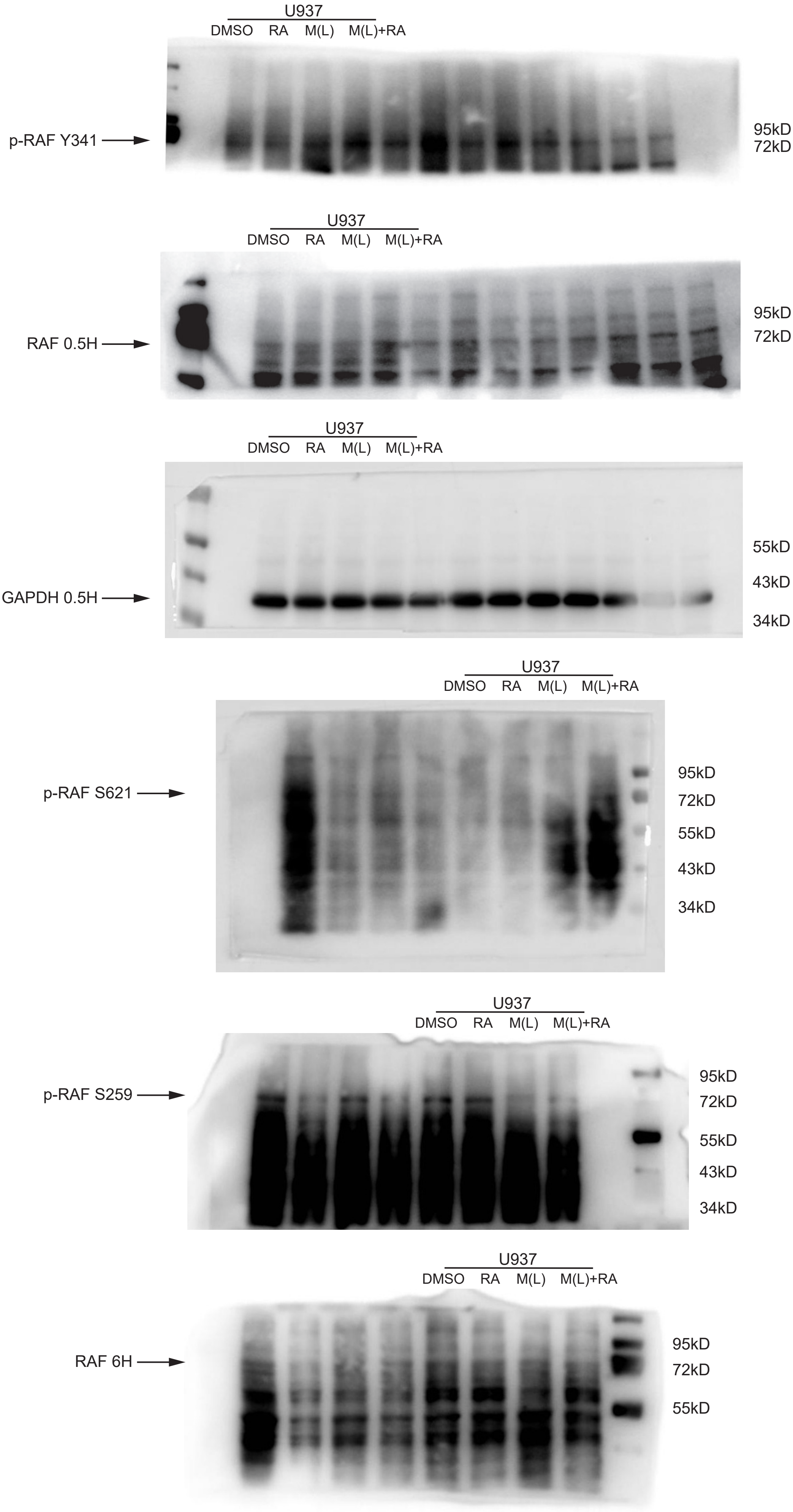
U937
DMSO RA M(L) M(L)+RA
p-RAF Y341
95kD
72kD
U937
DMSO RA M(L) M(L)+RA
RAF 0.5H
95kD
72kD
U937
DMSO RA M(L) M(L)+RA
GAPDH 0.5H
55kD
43kD
34kD
U937
DMSO RA M(L) M(L)+RA
p-RAF S621
95kD
72kD
55kD
43kD
34kD
U937
DMSO RA M(L) M(L)+RA
p-RAF S259
95kD
72kD
55kD
43kD
34kD
U937
DMSO RA M(L) M(L)+RA
RAF 6H
95kD
72kD
55kD

2A

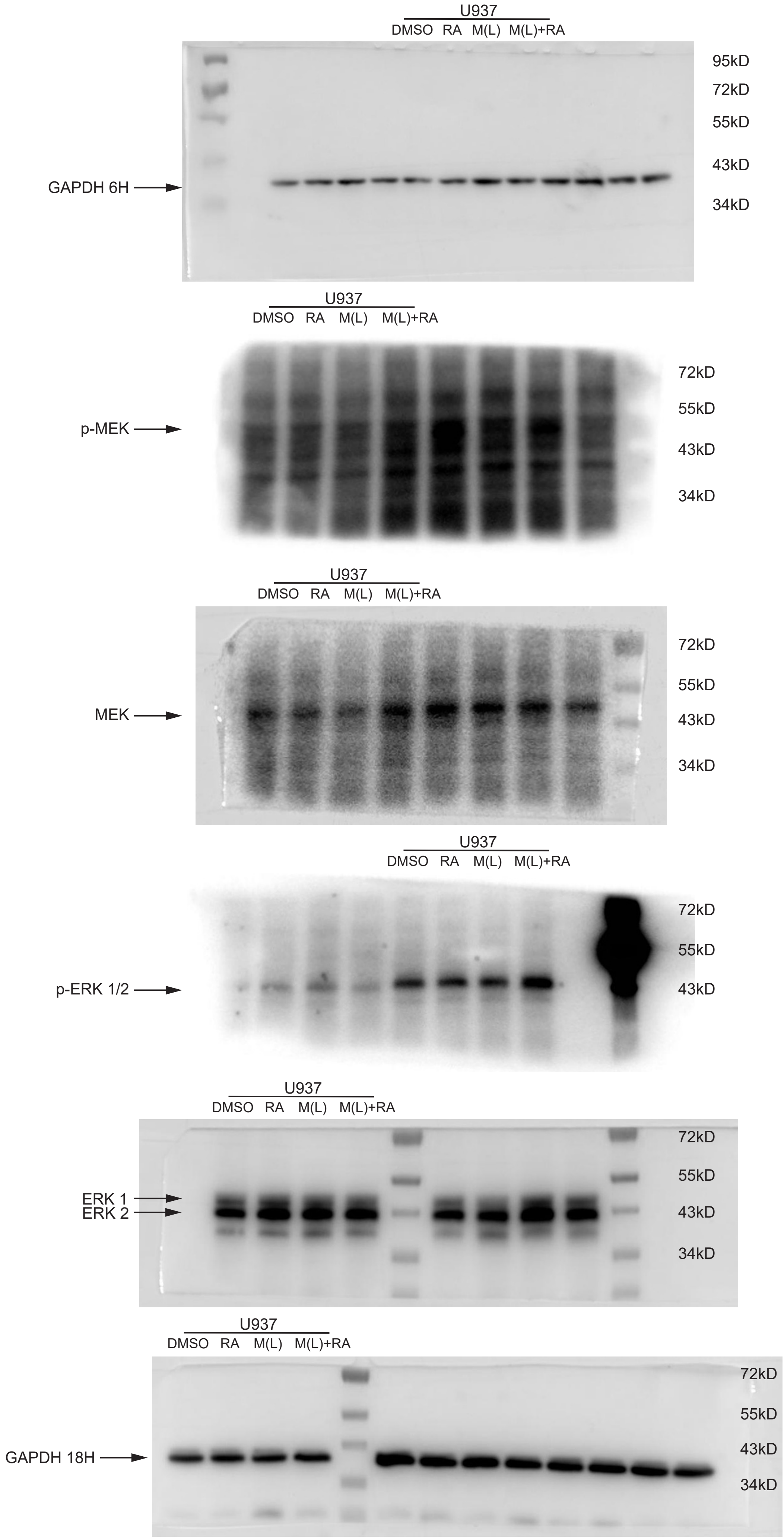
U937
DMSO RA M(L) M(L)+RA
95kD
72kD
55kD
43kD
34kD
GAPDH 6H
U937
DMSO RA M(L) M(L)+RA
72kD
55kD
43kD
34kD
p-MEK
U937
DMSO RA M(L) M(L)+RA
72kD
55kD
43kD
34kD
MEK
U937
DMSO RA M(L) M(L)+RA
72kD
55kD
43kD
p-ERK 1/2
U937
DMSO RA M(L) M(L)+RA
72kD
55kD
43kD
34kD
ERK 1
ERK 2
U937
DMSO RA M(L) M(L)+RA
72kD
55kD
43kD
34kD
GAPDH 18H

2A

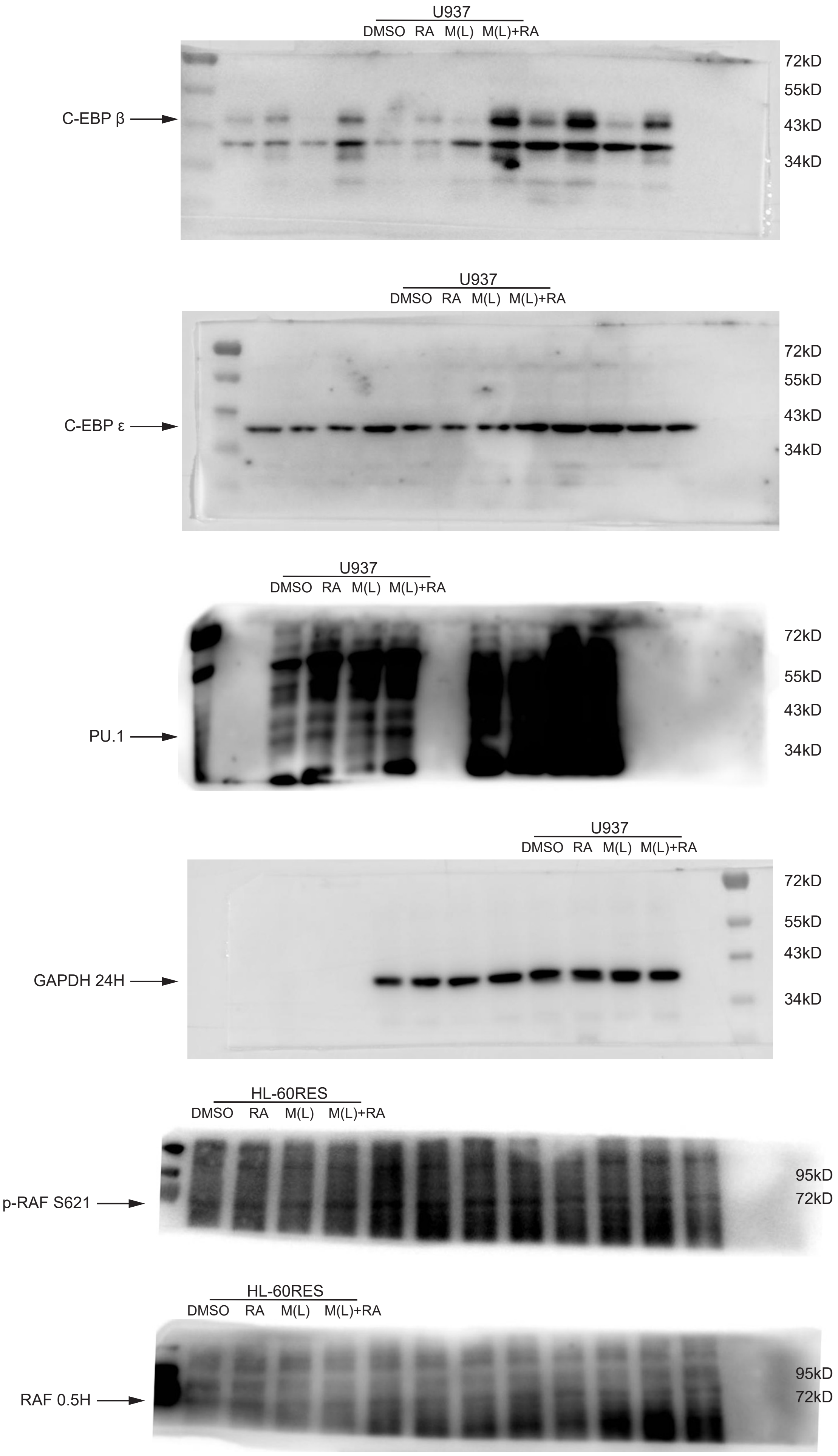
U937
DMSO RA M(L) M(L)+RA
C-EBP β
72kD
55kD
43kD
34kD
U937
DMSO RA M(L) M(L)+RA
C-EBP ε
72kD
55kD
43kD
34kD
U937
DMSO RA M(L) M(L)+RA
PU.1
72kD
55kD
43kD
34kD
U937
DMSO RA M(L) M(L)+RA
GAPDH 24H
72kD
55kD
43kD
34kD
HL-60RES
DMSO RA M(L) M(L)+RA
p-RAF S621
95kD
72kD
HL-60RES
DMSO RA M(L) M(L)+RA
RAF 0.5H
95kD
72kD

2A

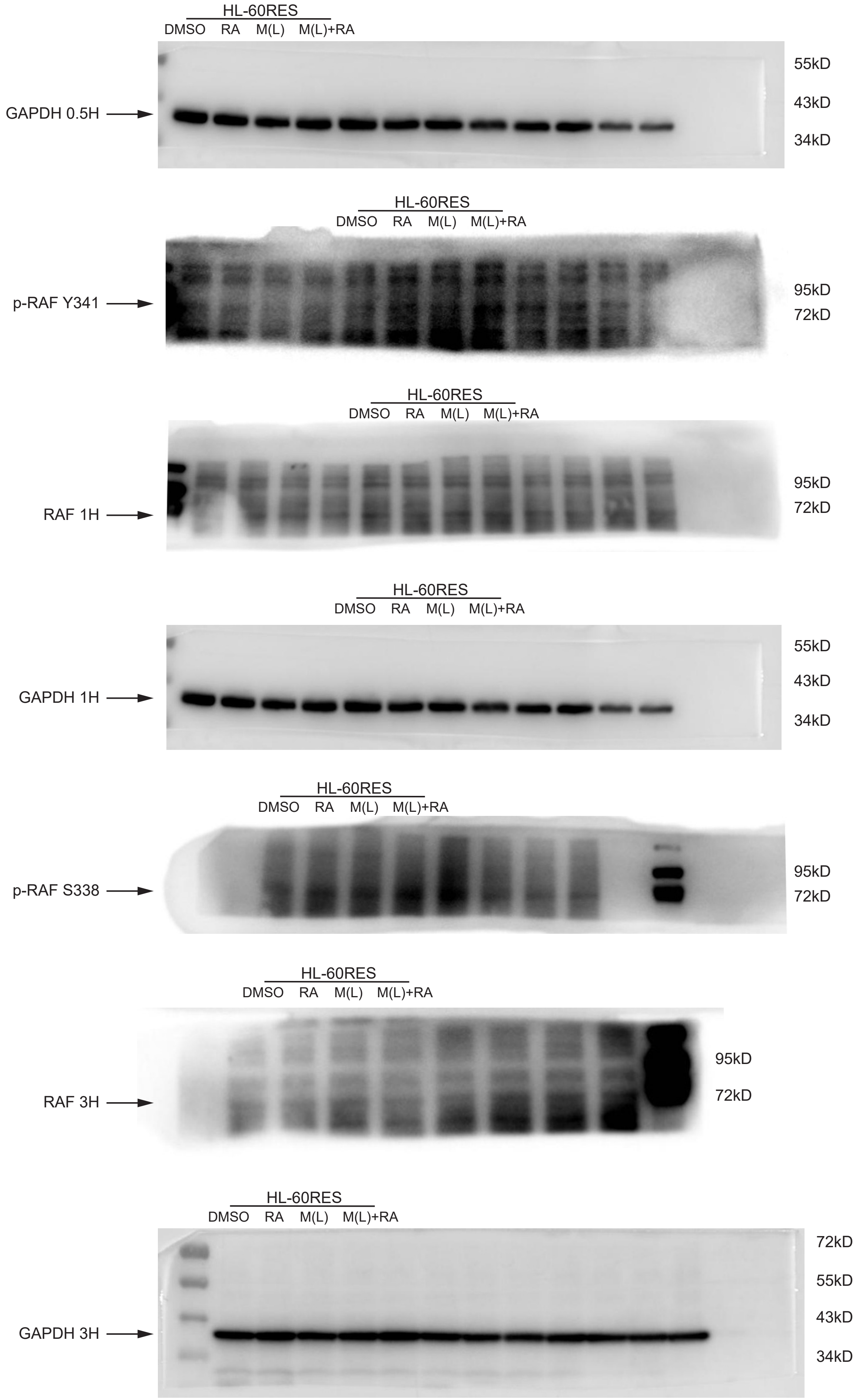

HL-60RES
DMSO RA M(L) M(L)+RA
GAPDH 0.5H
55kD
43kD
34kD
p-RAF Y341
95kD
72kD
RAF 1H
GAPDH 1H
p-RAF S338
RAF 3H
GAPDH 3H

2A

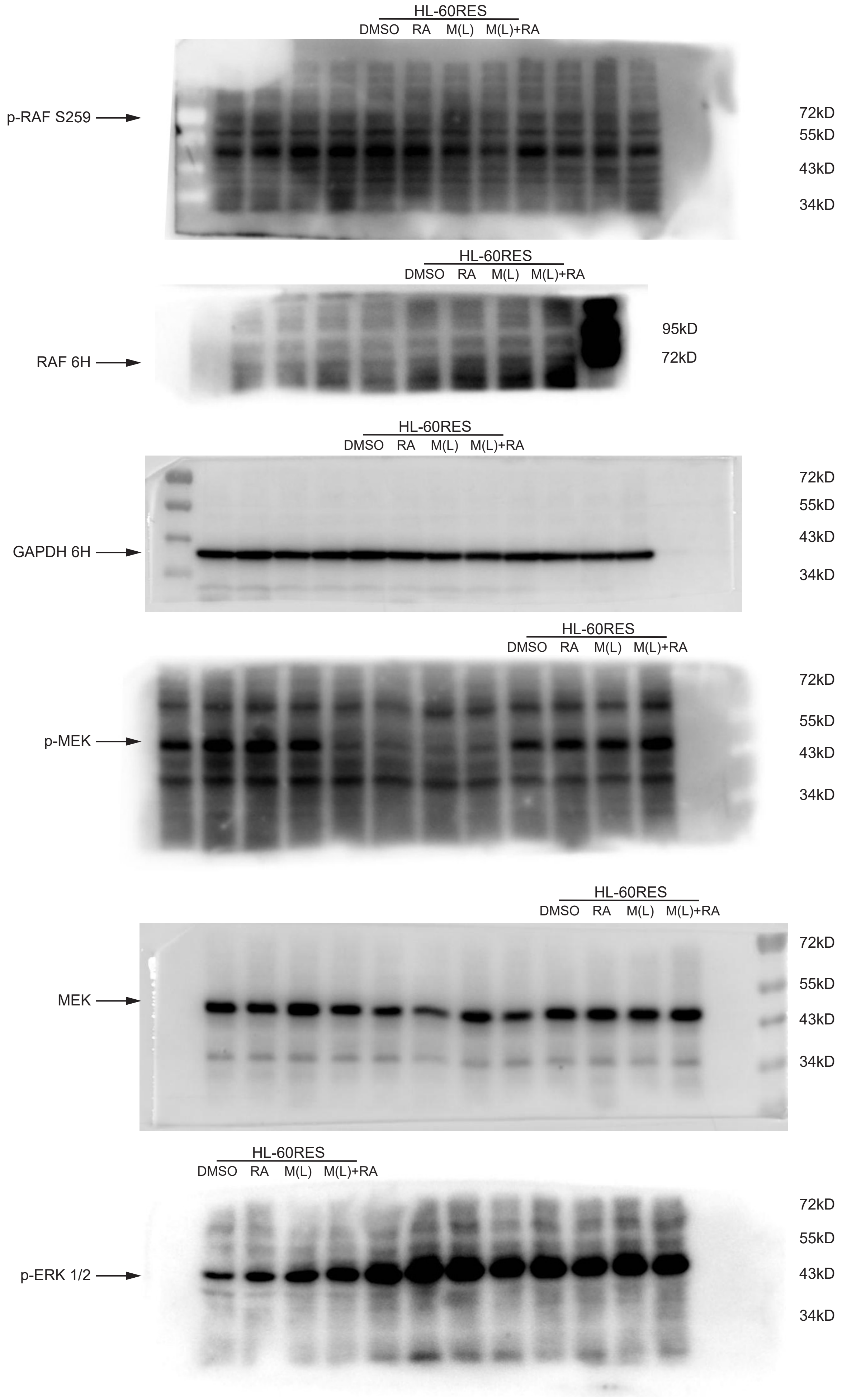
HL-60RES
DMSO RA M(L) M(L)+RA
p-RAF S259
72kD
55kD
43kD
34kD
HL-60RES
DMSO RA M(L) M(L)+RA
RAF 6H
95kD
72kD
HL-60RES
DMSO RA M(L) M(L)+RA
GAPDH 6H
72kD
55kD
43kD
34kD
HL-60RES
DMSO RA M(L) M(L)+RA
p-MEK
72kD
55kD
43kD
34kD
HL-60RES
DMSO RA M(L) M(L)+RA
MEK
72kD
55kD
43kD
34kD
HL-60RES
DMSO RA M(L) M(L)+RA
p-ERK 1/2
72kD
55kD
43kD
34kD

2A

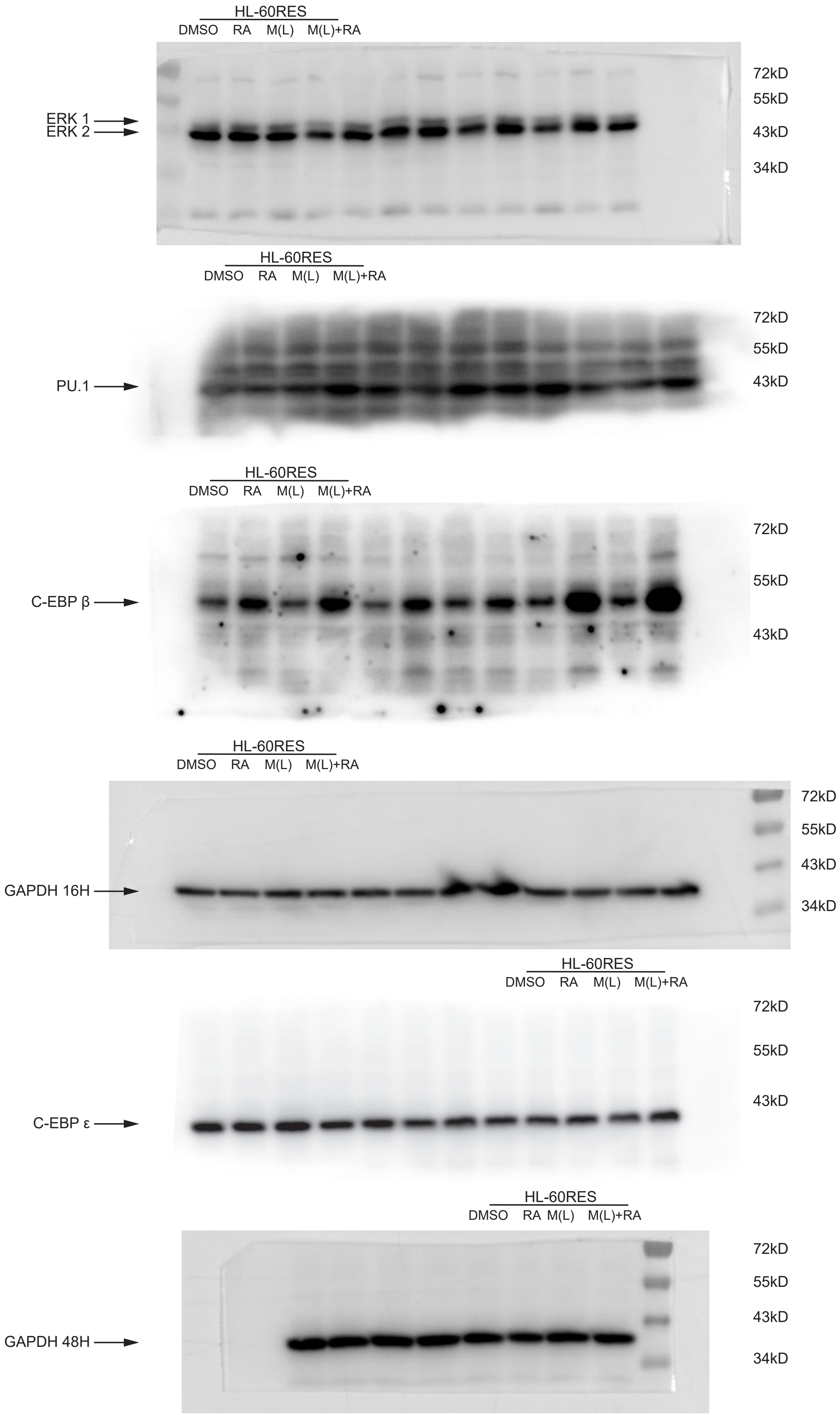
HL-60RES
DMSO RA M(L) M(L)+RA
ERK 1
ERK 2
72kD
55kD
43kD
34kD
PU.1
C-EBP β
GAPDH 16H
C-EBP ε
GAPDH 48H

2B

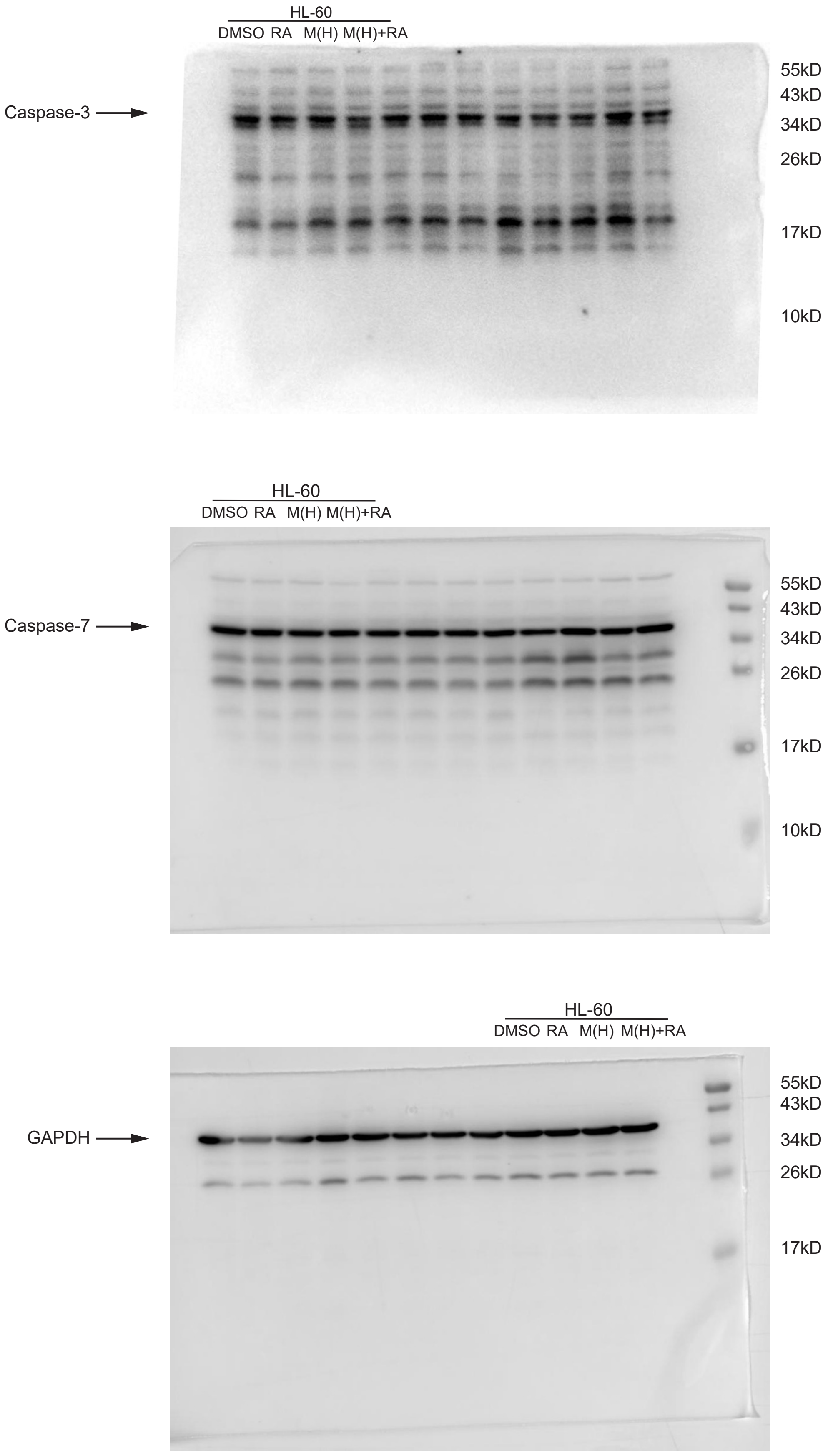
HL-60
DMSO RA M(H) M(H)+RA
Caspase-3
55kD
43kD
34kD
26kD
17kD
10kD
HL-60
DMSO RA M(H) M(H)+RA
Caspase-7
55kD
43kD
34kD
26kD
17kD
10kD
HL-60
DMSO RA M(H) M(H)+RA
GAPDH
55kD
43kD
34kD
26kD
17kD

2B

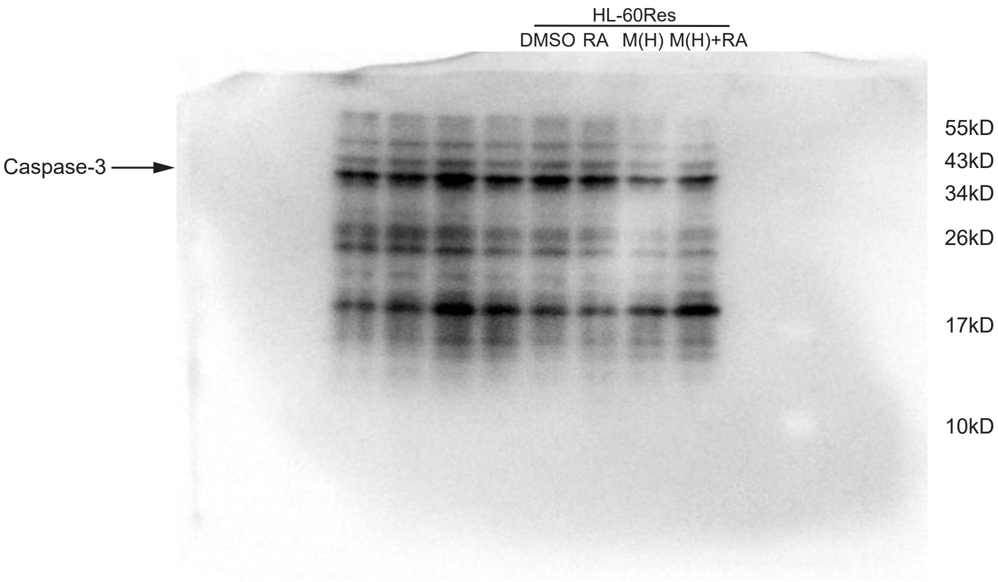
HL-60Res
DMSO RA M(H) M(H)+RA
Caspase-3
55kD
43kD
34kD
26kD
17kD
10kD

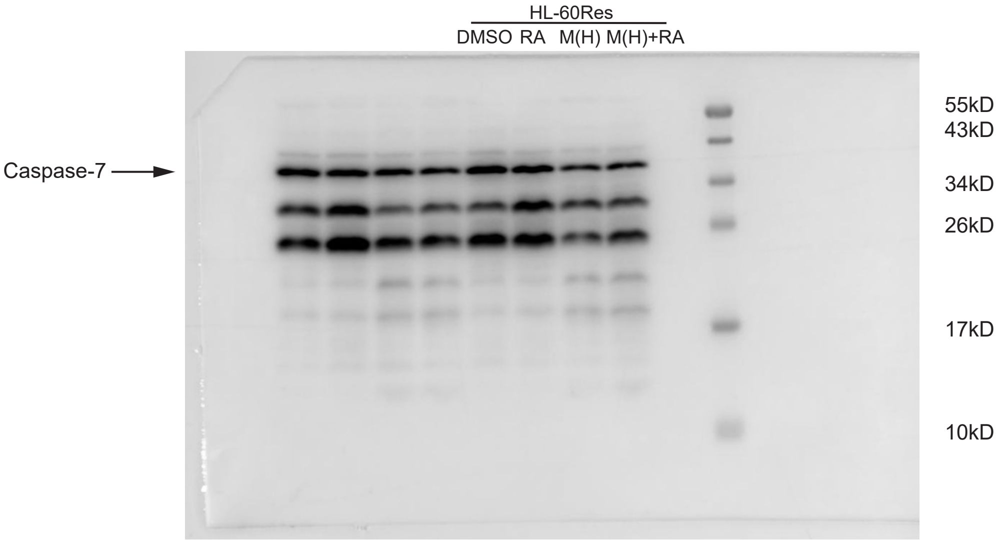
HL-60Res
DMSO RA M(H) M(H)+RA
Caspase-7
55kD
43kD
34kD
26kD
17kD
10kD

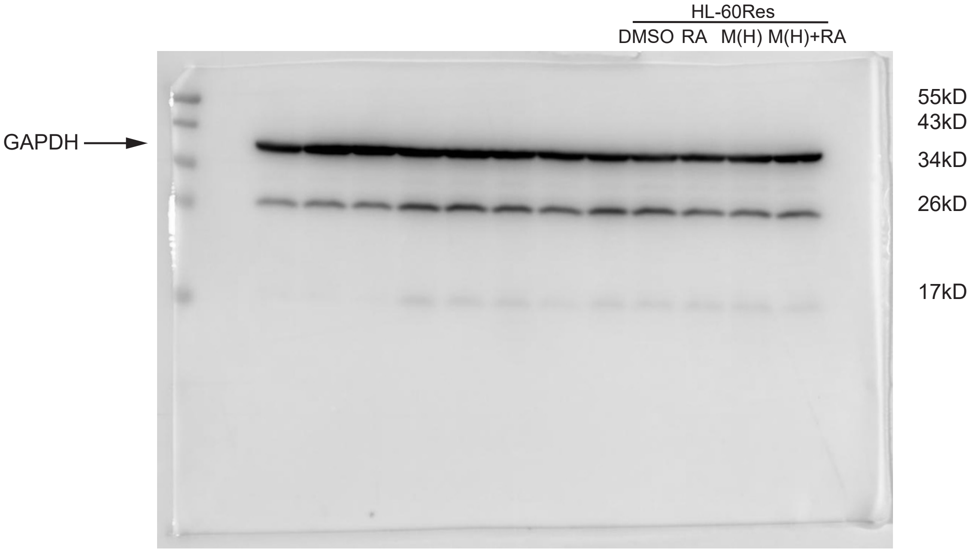
HL-60Res
DMSO RA M(H) M(H)+RA
GAPDH
55kD
43kD
34kD
26kD
17kD

2B

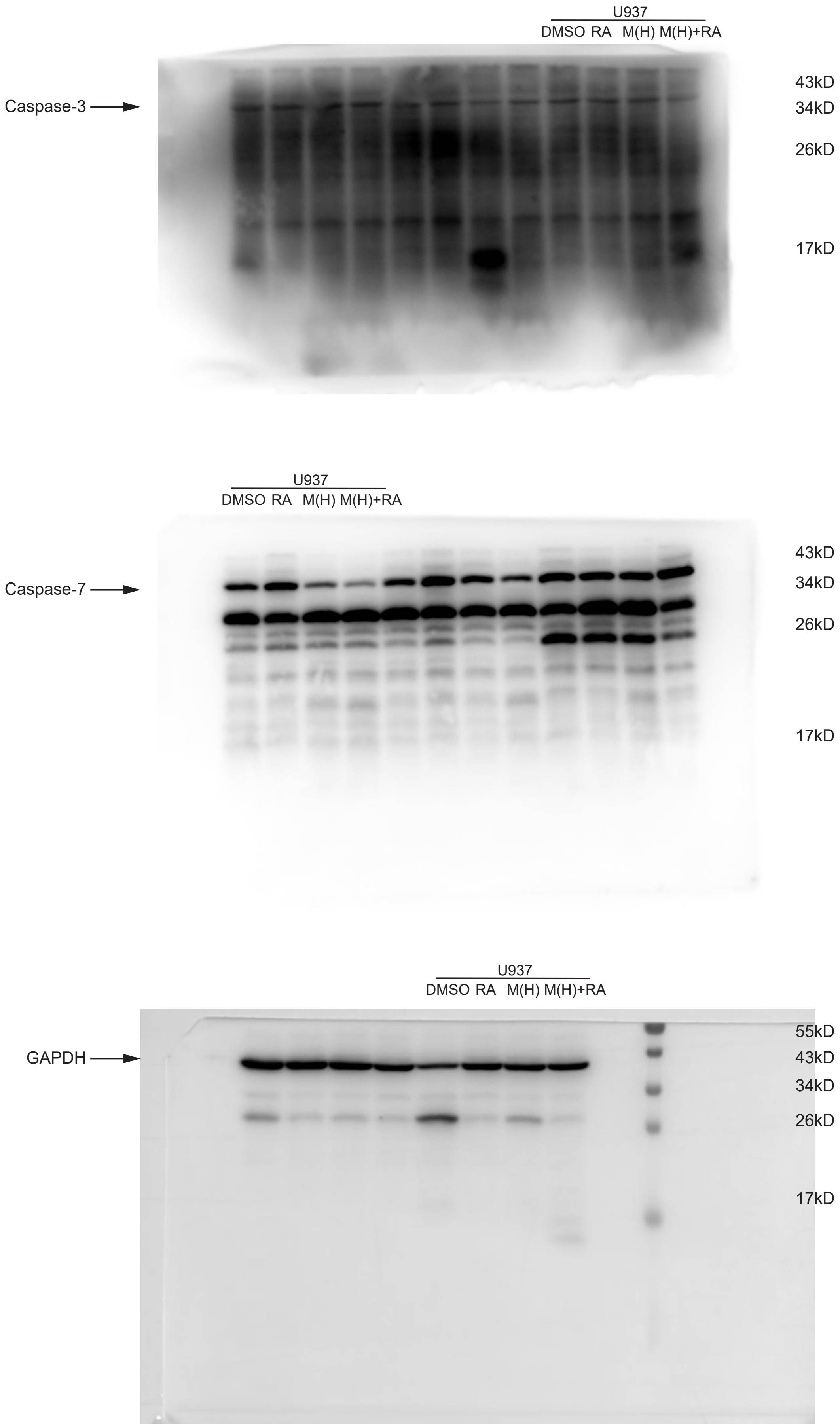
U937
DMSO RA M(H) M(H)+RA
Caspase-3
43kD
34kD
26kD
17kD
U937
DMSO RA M(H) M(H)+RA
Caspase-7
43kD
34kD
26kD
17kD
U937
DMSO RA M(H) M(H)+RA
GAPDH
55kD
43kD
34kD
26kD
17kD

3D

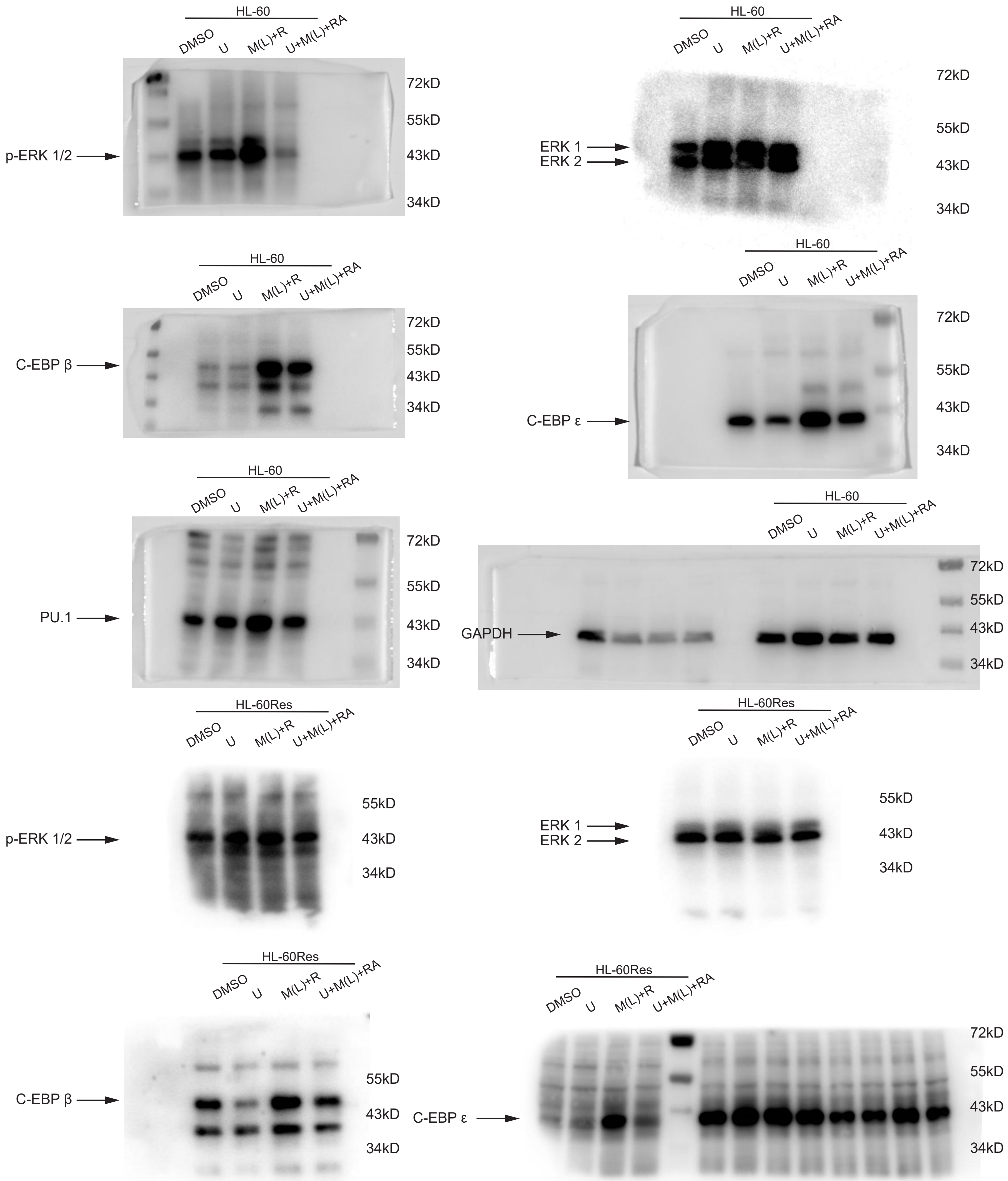

HL-60
DMSO
U
M(L)+R
U+M(L)+RA
72kD
55kD
43kD
34kD
p-ERK 1/2
ERK 1
ERK 2
C-EBP β
C-EBP ε
PU.1
GAPDH
HL-60Res

3D

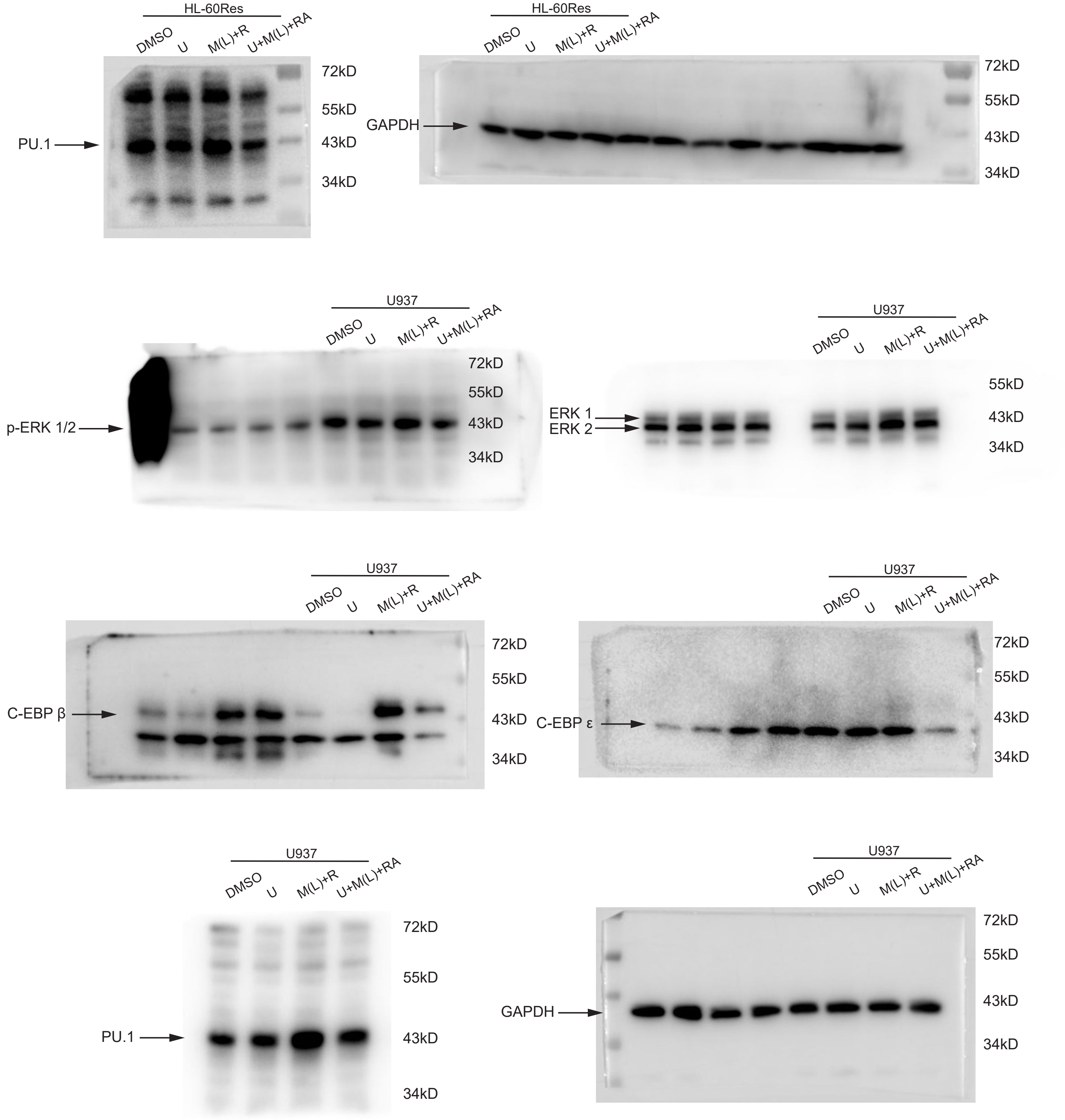

HL-60Res
DMSO
U
M(L)+R
U+M(L)+RA
72kD
55kD
43kD
34kD
PU.1
GAPDH
U937
p-ERK 1/2
ERK 1
ERK 2
C-EBP β
C-EBP ε

4A

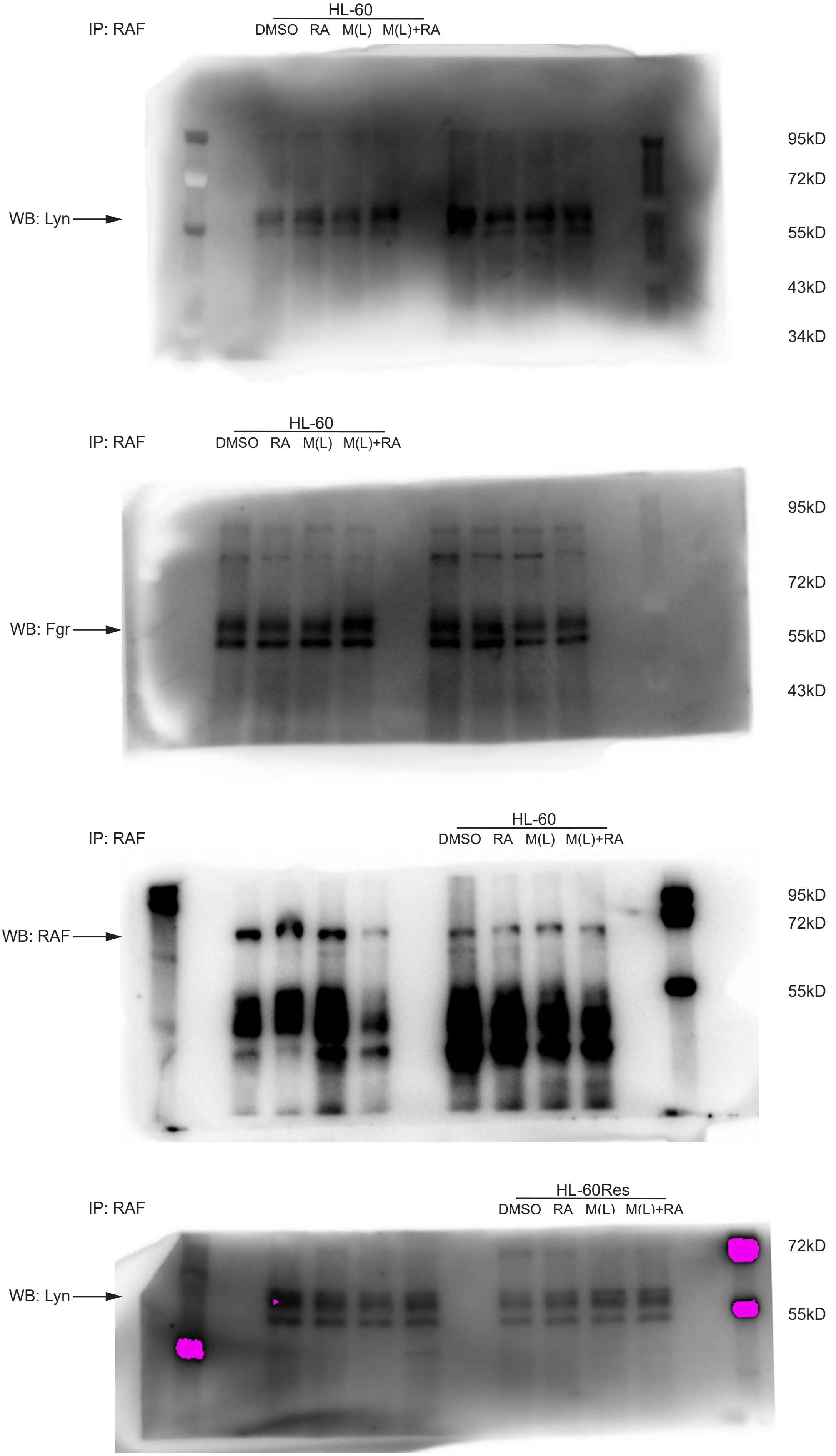
IP: RAF
HL-60
DMSO RA M(L) M(L)+RA
WB: Lyn
95kD
72kD
55kD
43kD
34kD
IP: RAF
HL-60
DMSO RA M(L) M(L)+RA
WB: Fgr
95kD
72kD
55kD
43kD
IP: RAF
HL-60
DMSO RA M(L) M(L)+RA
WB: RAF
95kD
72kD
55kD
IP: RAF
HL-60Res
DMSO RA M(L) M(L)+RA
WB: Lyn
72kD
55kD

4A

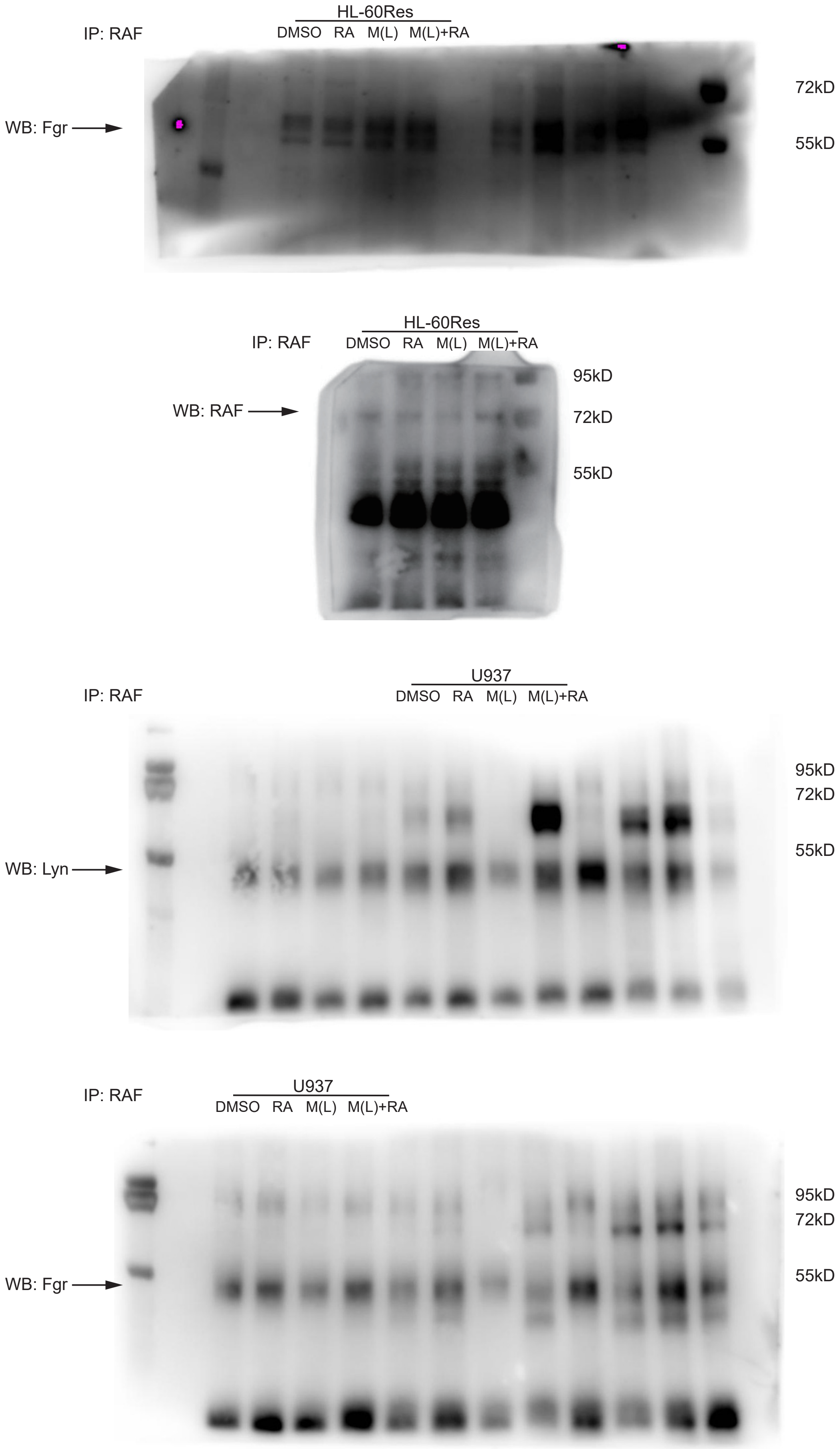
HL-60Res
IP: RAF
DMSO RA M(L) M(L)+RA
WB: Fgr
72kD
55kD
HL-60Res
IP: RAF
DMSO RA M(L) M(L)+RA
WB: RAF
95kD
72kD
55kD
U937
IP: RAF
DMSO RA M(L) M(L)+RA
WB: Lyn
95kD
72kD
55kD
U937
IP: RAF
DMSO RA M(L) M(L)+RA
WB: Fgr
95kD
72kD
55kD

4A

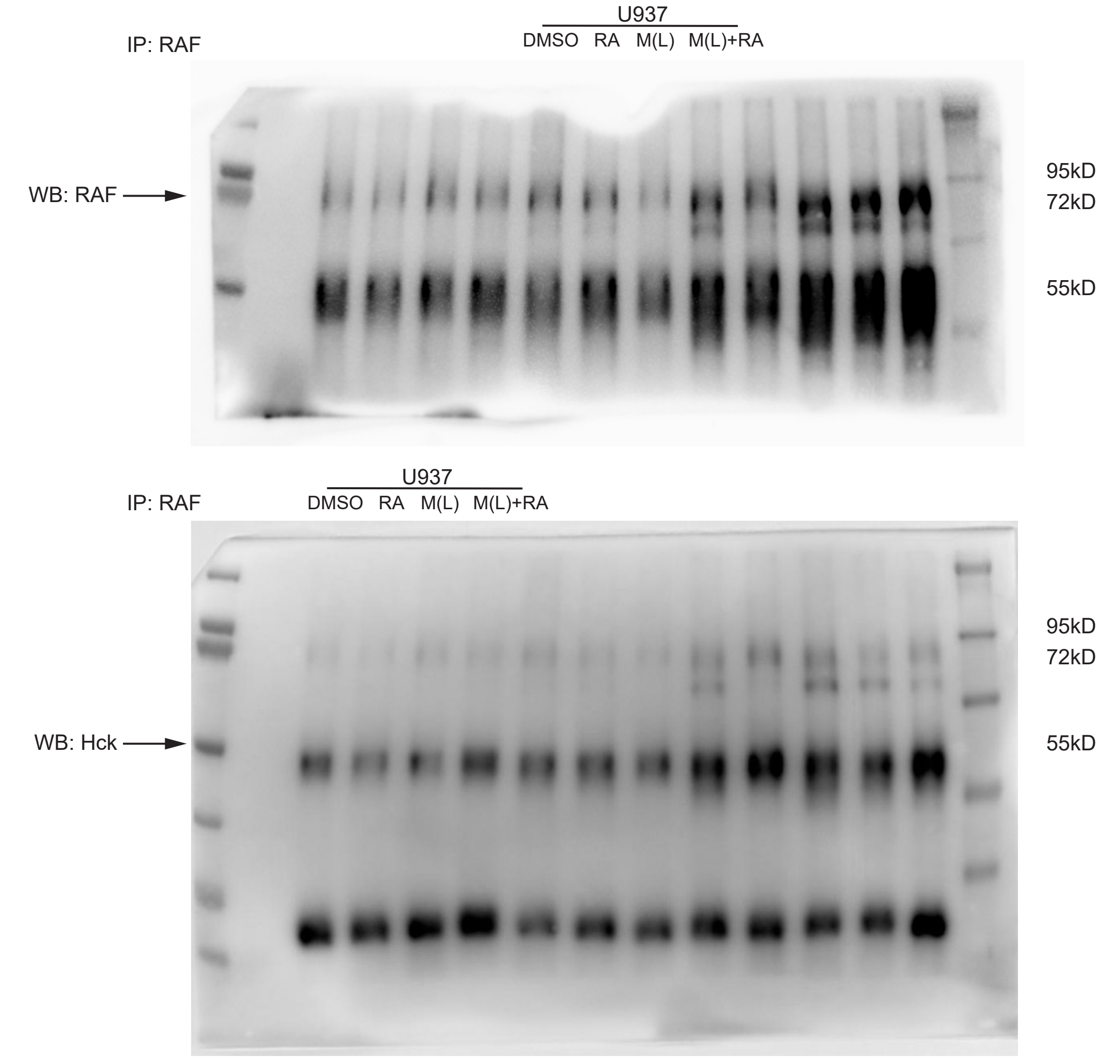
U937
DMSO RA M(L) M(L)+RA
IP: RAF
WB: RAF
95kD
72kD
55kD
U937
DMSO RA M(L) M(L)+RA
IP: RAF
WB: Hck
95kD
72kD
55kD

4B

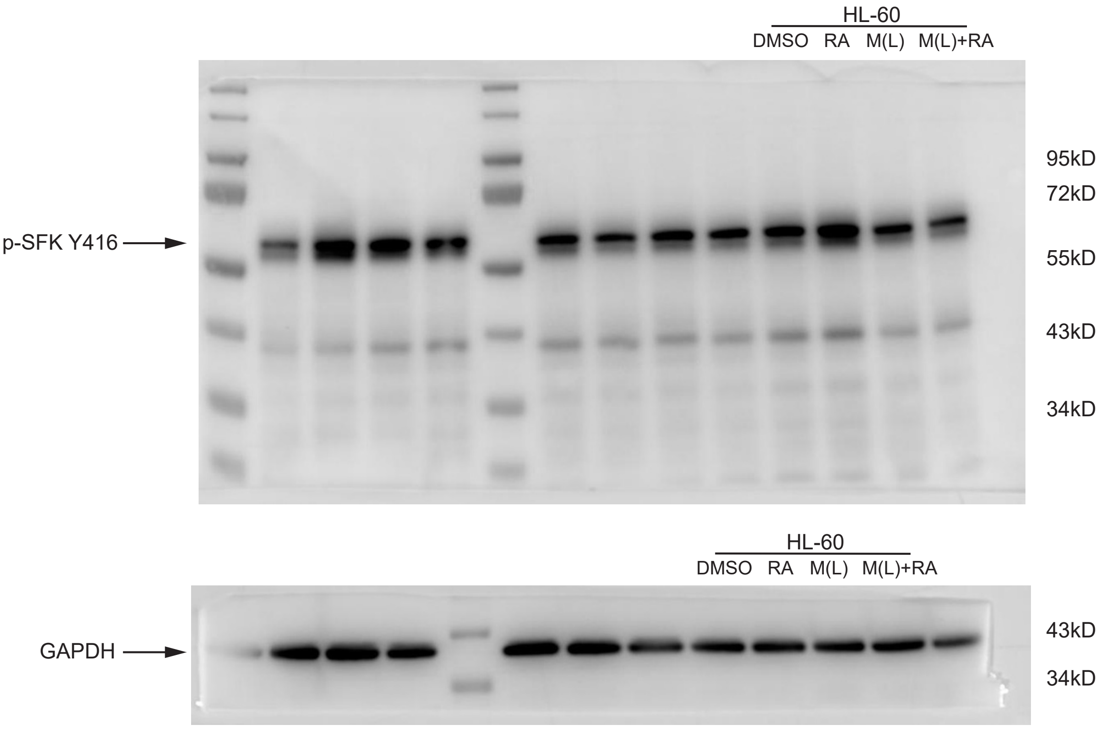
HL-60
DMSO RA M(L) M(L)+RA
p-SFK Y416
95kD
72kD
55kD
43kD
34kD
HL-60
DMSO RA M(L) M(L)+RA
GAPDH
43kD
34kD

4B

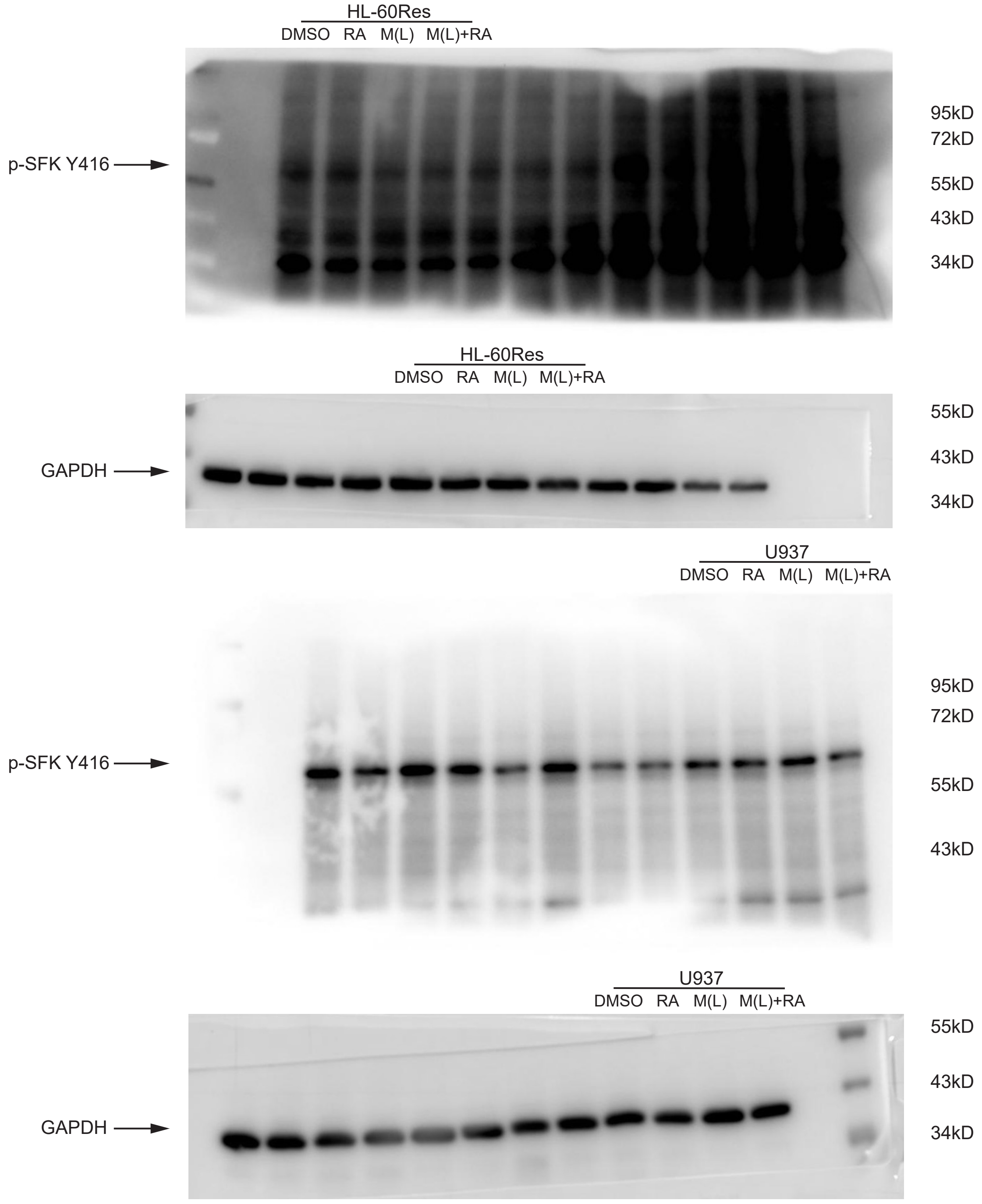
HL-60Res
DMSO RA M(L) M(L)+RA
p-SFK Y416
95kD
72kD
55kD
43kD
34kD
HL-60Res
DMSO RA M(L) M(L)+RA
GAPDH
55kD
43kD
34kD
U937
DMSO RA M(L) M(L)+RA
p-SFK Y416
95kD
72kD
55kD
43kD
U937
DMSO RA M(L) M(L)+RA
GAPDH
55kD
43kD
34kD

4C

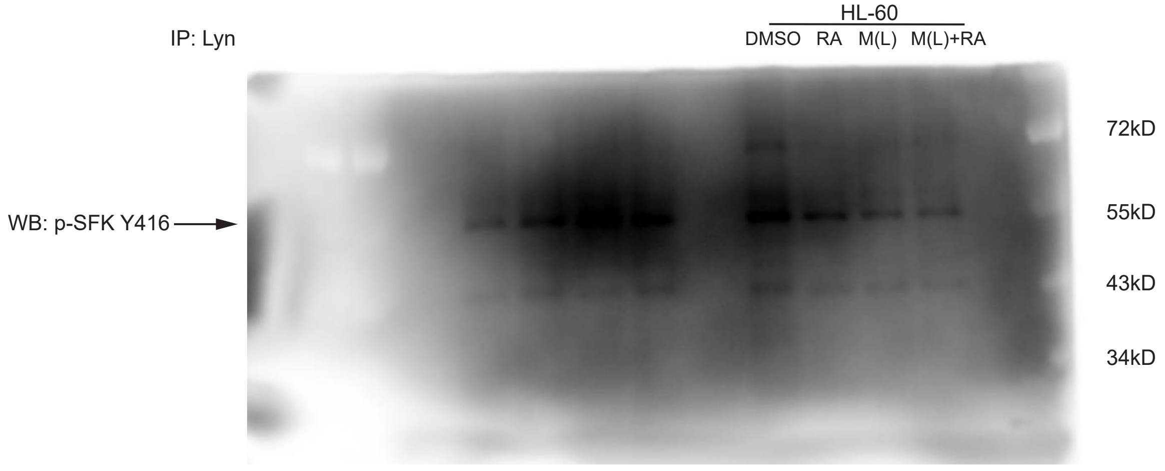
IP: Lyn
HL-60
DMSO RA M(L) M(L)+RA
WB: p-SFK Y416
72kD
55kD
43kD
34kD

4C

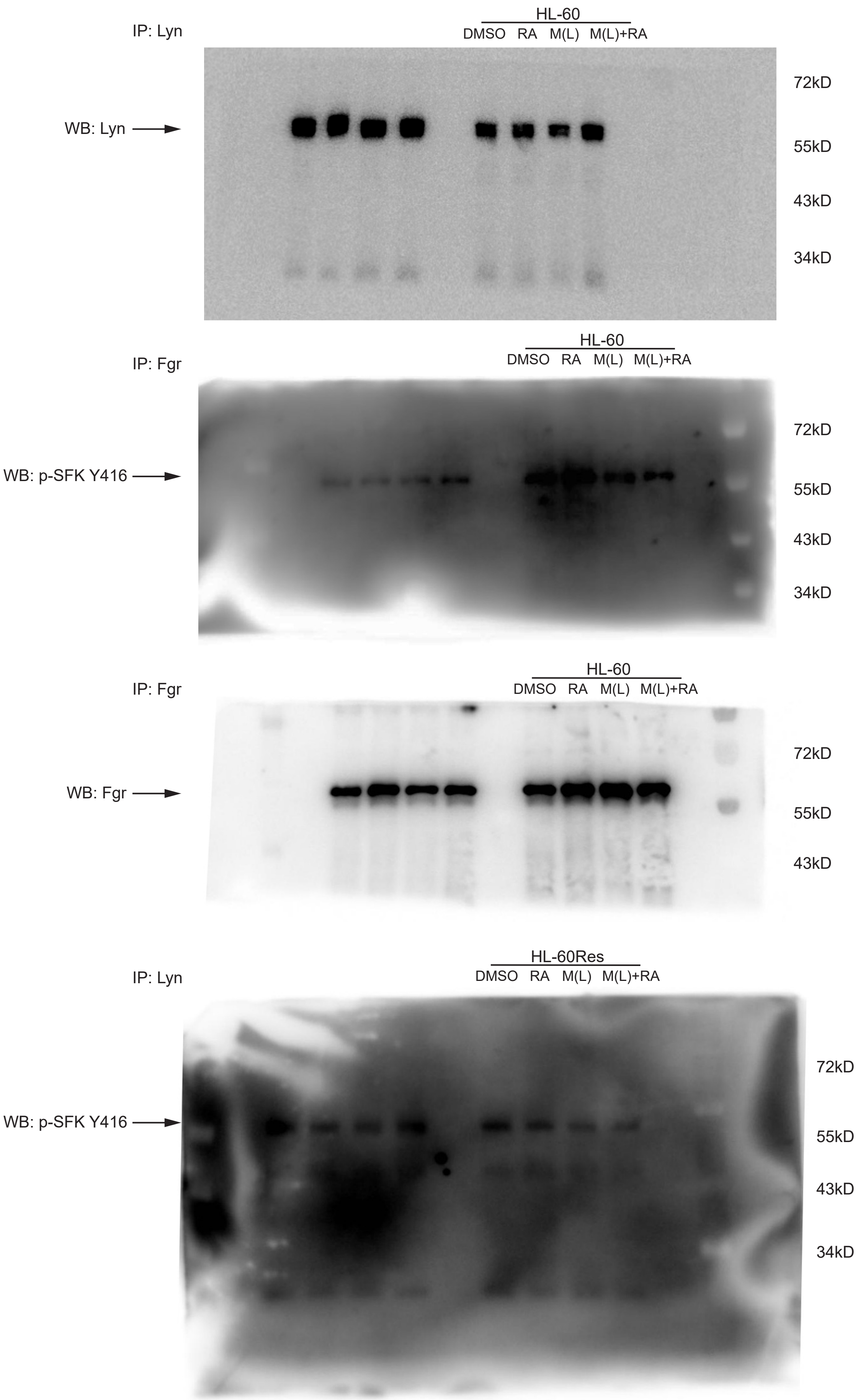
IP: Lyn
HL-60
DMSO RA M(L) M(L)+RA
WB: Lyn
72kD
55kD
43kD
34kD
IP: Fgr
HL-60
DMSO RA M(L) M(L)+RA
WB: p-SFK Y416
72kD
55kD
43kD
34kD
IP: Fgr
HL-60
DMSO RA M(L) M(L)+RA
WB: Fgr
72kD
55kD
43kD
IP: Lyn
HL-60Res
DMSO RA M(L) M(L)+RA
WB: p-SFK Y416
72kD
55kD
43kD
34kD

4C

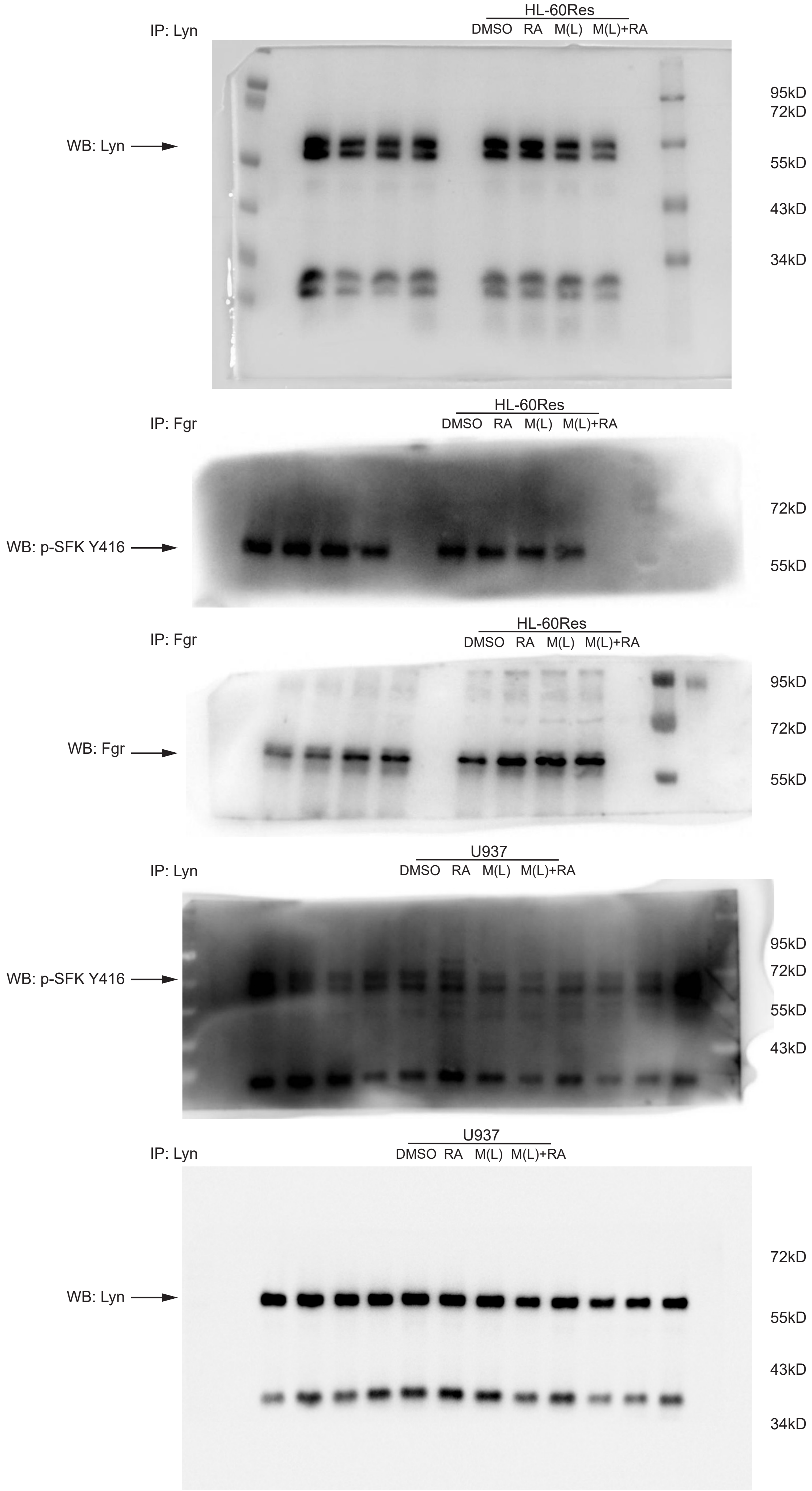
HL-60Res
IP: Lyn
DMSO RA M(L) M(L)+RA
95kD
72kD
WB: Lyn
55kD
43kD
34kD
HL-60Res
IP: Fgr
DMSO RA M(L) M(L)+RA
72kD
WB: p-SFK Y416
55kD
HL-60Res
IP: Fgr
DMSO RA M(L) M(L)+RA
95kD
72kD
WB: Fgr
55kD
U937
IP: Lyn
DMSO RA M(L) M(L)+RA
95kD
72kD
WB: p-SFK Y416
55kD
43kD
U937
IP: Lyn
DMSO RA M(L) M(L)+RA
72kD
WB: Lyn
55kD
43kD
34kD

4C

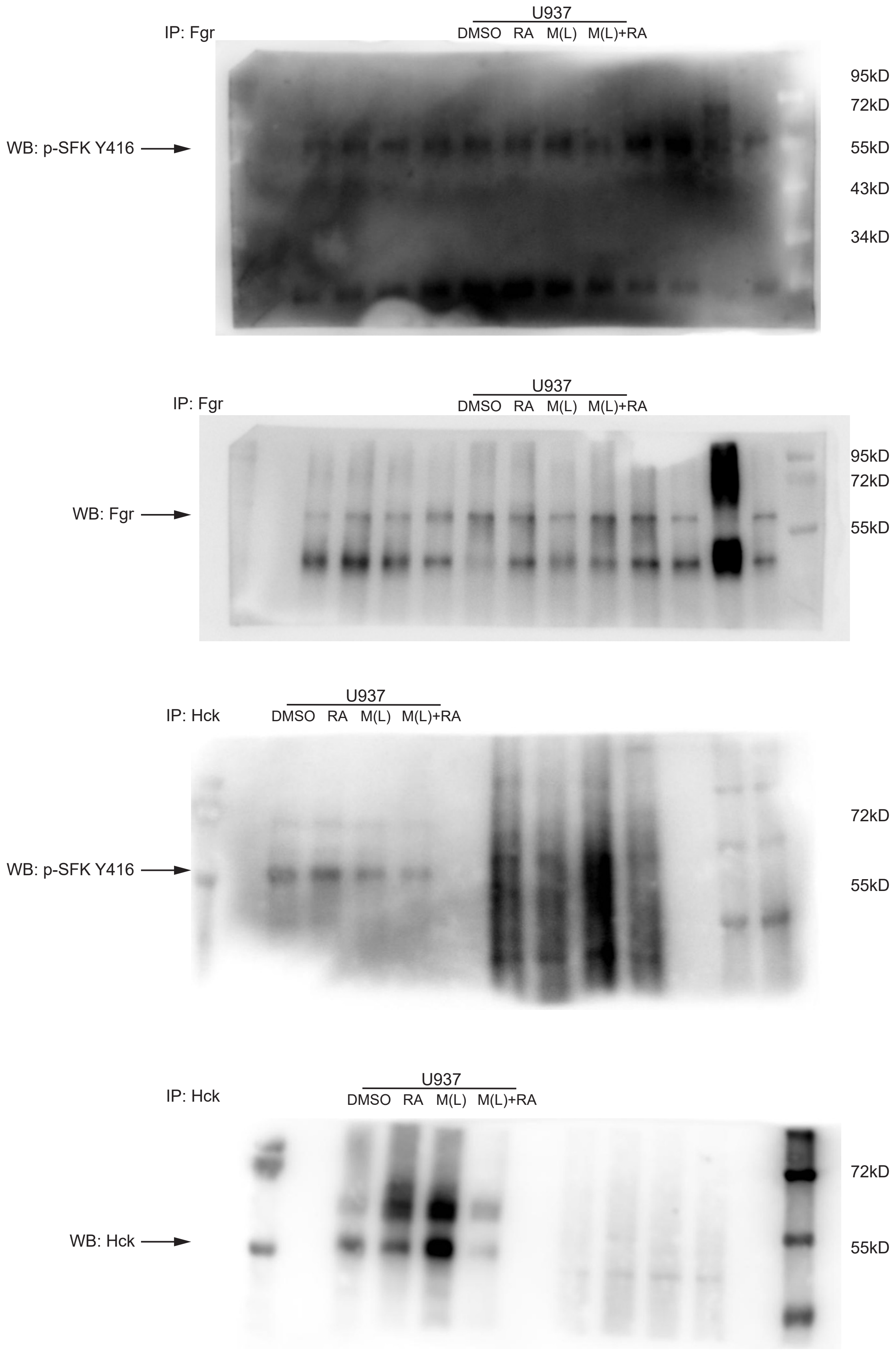
U937
IP: Fgr
DMSO RA M(L) M(L)+RA
95kD
72kD
WB: p-SFK Y416
55kD
43kD
34kD
U937
IP: Fgr
DMSO RA M(L) M(L)+RA
95kD
72kD
WB: Fgr
55kD
U937
IP: Hck
DMSO RA M(L) M(L)+RA
72kD
WB: p-SFK Y416
55kD
U937
IP: Hck
DMSO RA M(L) M(L)+RA
72kD
WB: Hck
55kD

5A

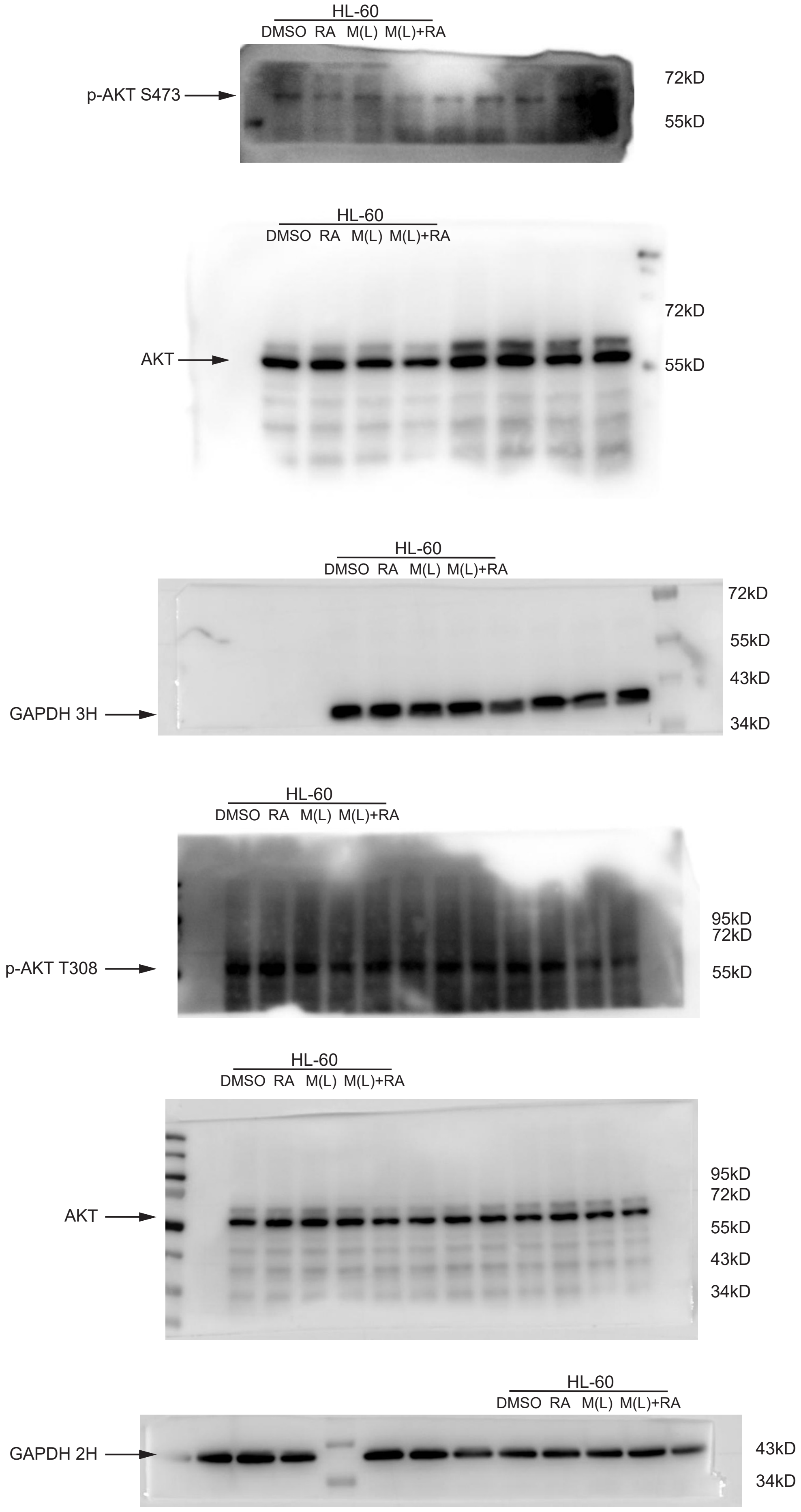

HL-60
DMSO RA M(L) M(L)+RA
p-AKT S473
72kD
55kD
HL-60
DMSO RA M(L) M(L)+RA
AKT
72kD
55kD
HL-60
DMSO RA M(L) M(L)+RA
GAPDH 3H
72kD
55kD
43kD
34kD
HL-60
DMSO RA M(L) M(L)+RA
p-AKT T308
95kD
72kD
55kD
HL-60
DMSO RA M(L) M(L)+RA
AKT
95kD
72kD
55kD
43kD
34kD
HL-60
DMSO RA M(L) M(L)+RA
GAPDH 2H
43kD
34kD

5A

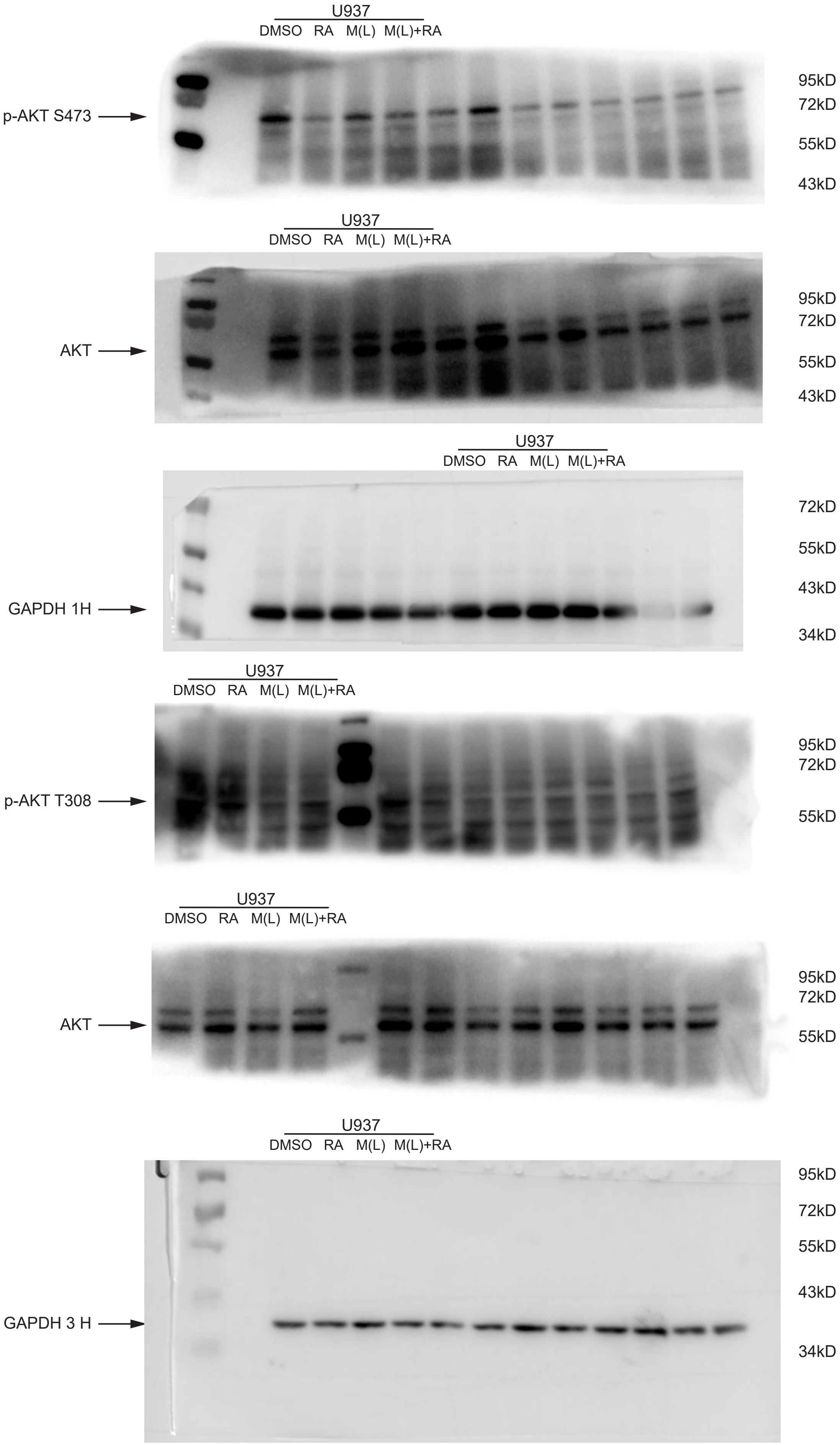
U937
DMSO RA M(L) M(L)+RA
p-AKT S473
95kD
72kD
55kD
43kD
U937
DMSO RA M(L) M(L)+RA
AKT
95kD
72kD
55kD
43kD
U937
DMSO RA M(L) M(L)+RA
GAPDH 1H
72kD
55kD
43kD
34kD
U937
DMSO RA M(L) M(L)+RA
p-AKT T308
95kD
72kD
55kD
U937
DMSO RA M(L) M(L)+RA
AKT
95kD
72kD
55kD
U937
DMSO RA M(L) M(L)+RA
GAPDH 3 H
95kD
72kD
55kD
43kD
34kD

5A

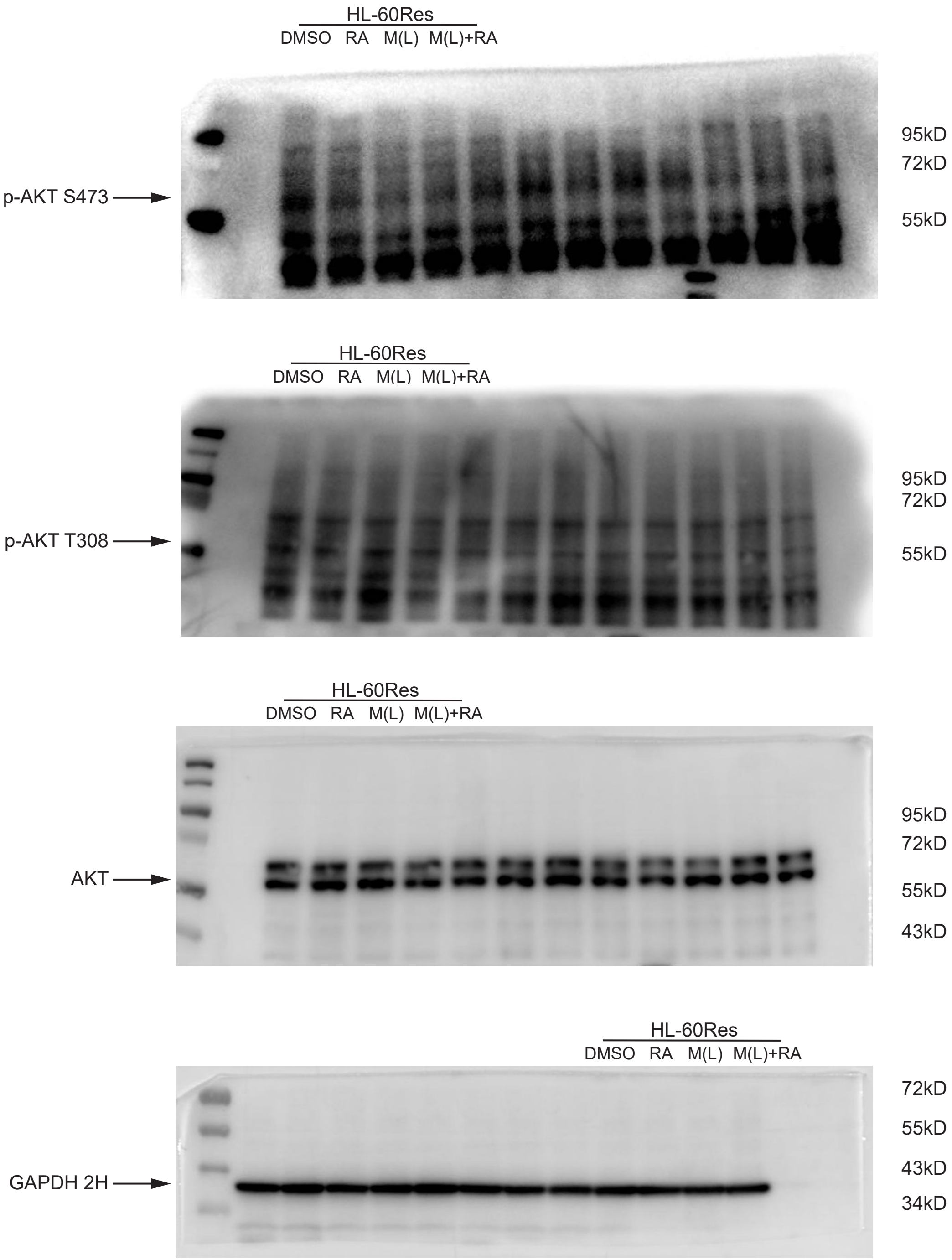
HL-60Res
DMSO RA M(L) M(L)+RA
p-AKT S473
95kD
72kD
55kD
HL-60Res
DMSO RA M(L) M(L)+RA
p-AKT T308
95kD
72kD
55kD
HL-60Res
DMSO RA M(L) M(L)+RA
AKT
95kD
72kD
55kD
43kD
HL-60Res
DMSO RA M(L) M(L)+RA
GAPDH 2H
72kD
55kD
43kD
34kD

5E

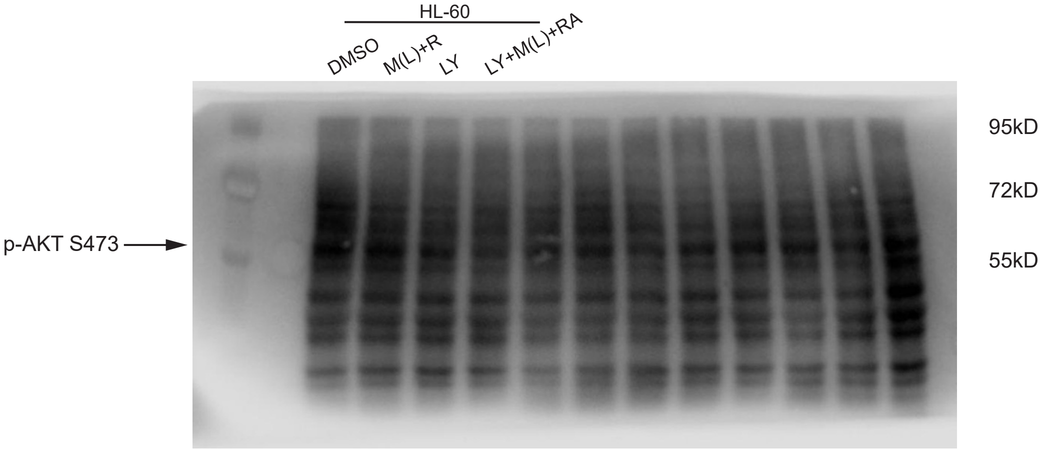
HL-60
DMSO
M(L)+R
LY
LY+M(L)+RA
p-AKT S473
95kD
72kD
55kD

5E

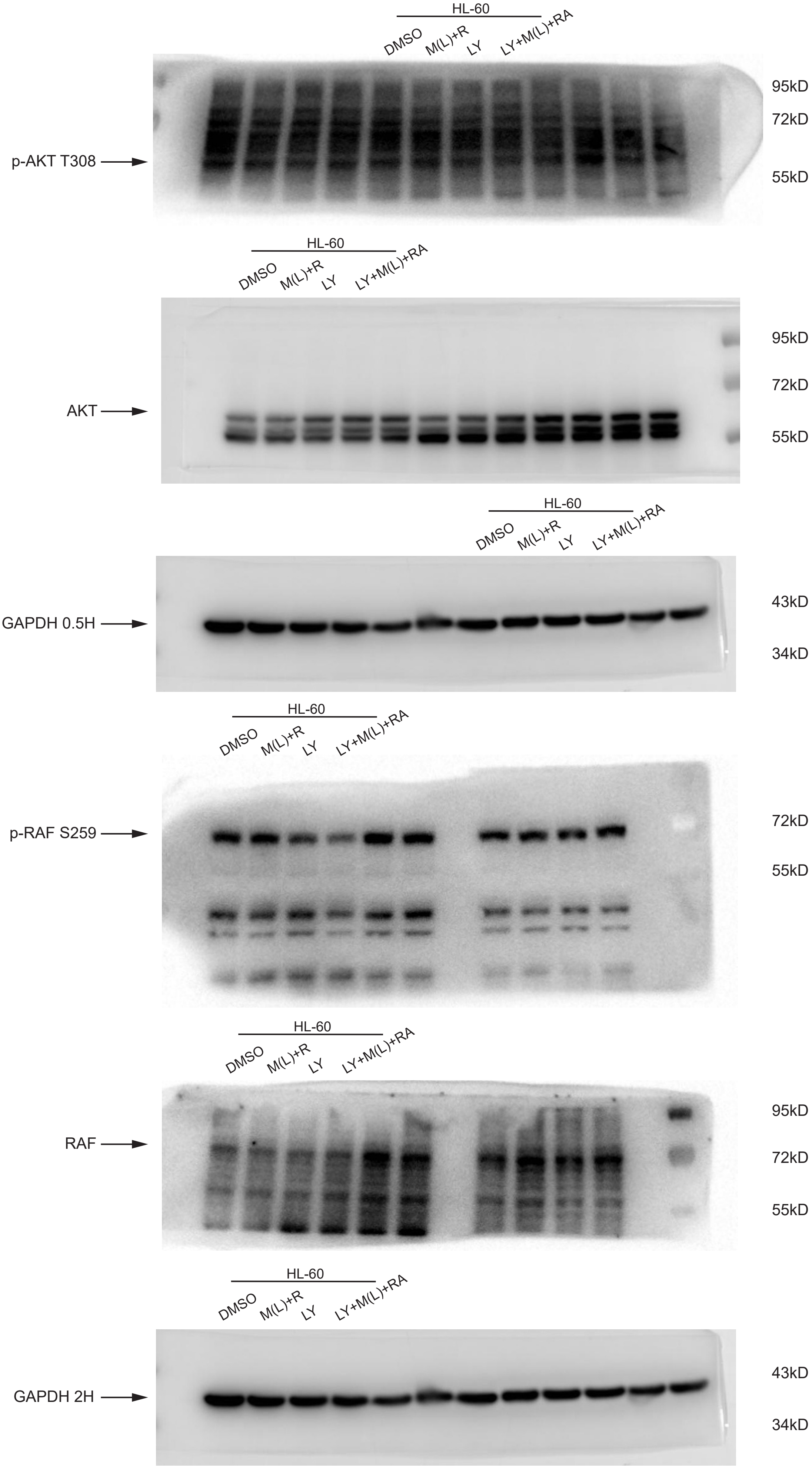
HL-60
DMSO
M(L)+R
LY
LY+M(L)+RA
p-AKT T308
95kD
72kD
55kD
HL-60
DMSO
M(L)+R
LY
LY+M(L)+RA
AKT
95kD
72kD
55kD
HL-60
DMSO
M(L)+R
LY
LY+M(L)+RA
GAPDH 0.5H
43kD
34kD
HL-60
DMSO
M(L)+R
LY
LY+M(L)+RA
p-RAF S259
72kD
55kD
HL-60
DMSO
M(L)+R
LY
LY+M(L)+RA
RAF
95kD
72kD
55kD
HL-60
DMSO
M(L)+R
LY
LY+M(L)+RA
GAPDH 2H
43kD
34kD

5E

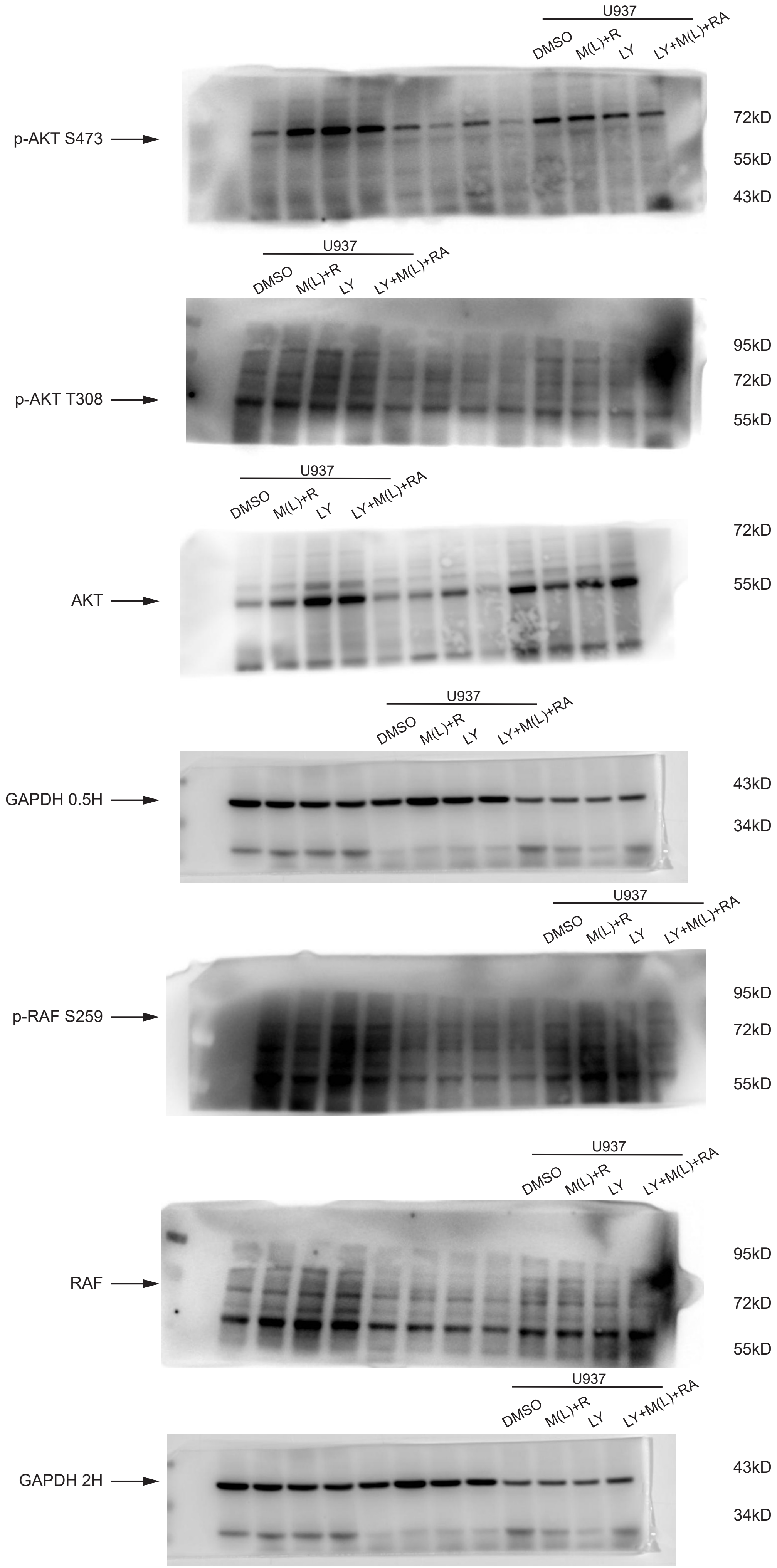
U937
DMSO
M(L)+R
LY
LY+M(L)+RA
p-AKT S473
72kD
55kD
43kD
p-AKT T308
95kD
72kD
55kD
AKT
72kD
55kD
GAPDH 0.5H
43kD
34kD
p-RAF S259
95kD
72kD
55kD
RAF
95kD
72kD
55kD
GAPDH 2H
43kD
34kD

5E

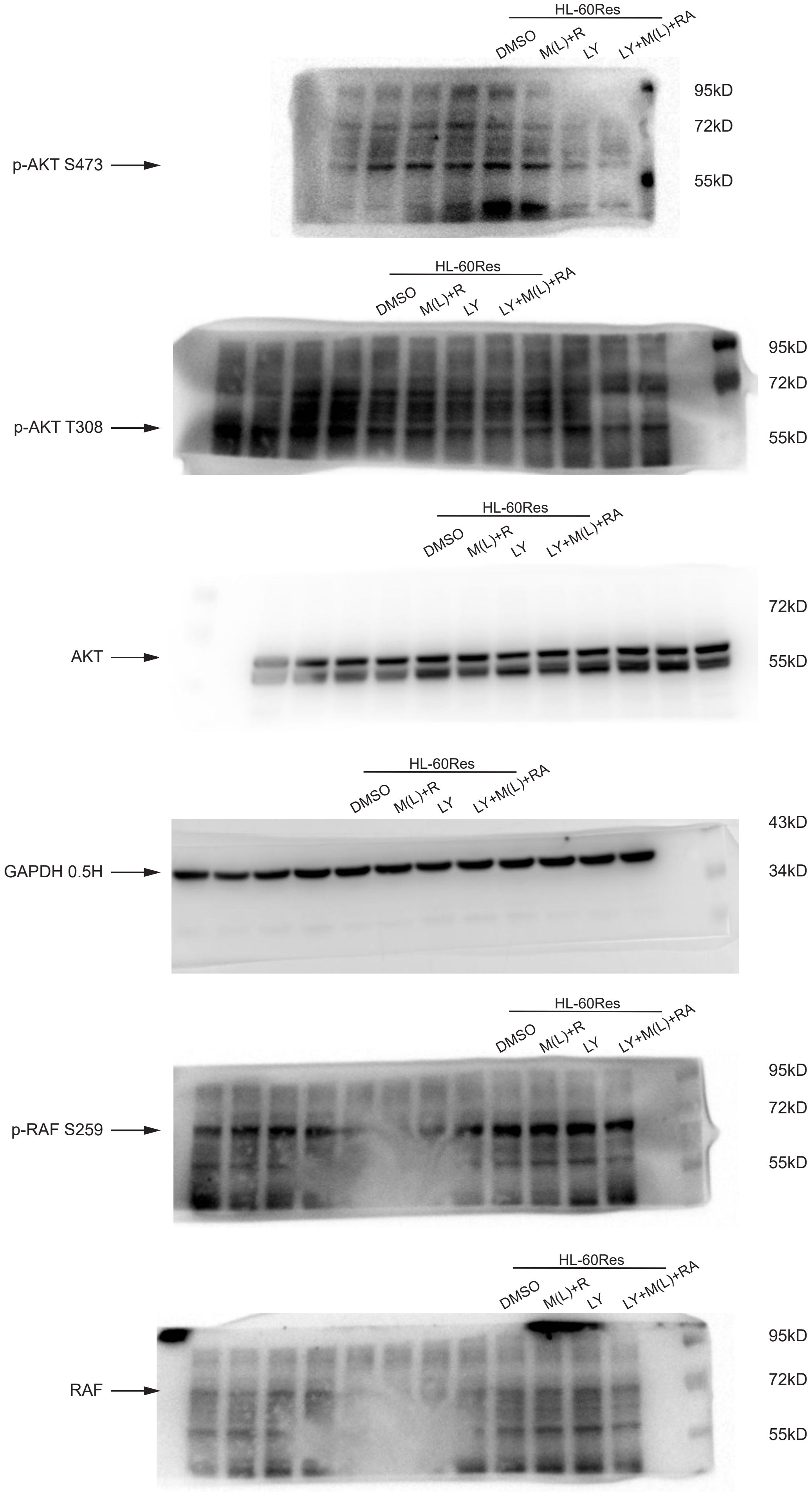

HL-60Res
DMSO
M(L)+R
LY
LY+M(L)+RA
p-AKT S473
95kD
72kD
55kD
HL-60Res
DMSO
M(L)+R
LY
LY+M(L)+RA
p-AKT T308
95kD
72kD
55kD
HL-60Res
DMSO
M(L)+R
LY
LY+M(L)+RA
AKT
72kD
55kD
HL-60Res
DMSO
M(L)+R
LY
LY+M(L)+RA
GAPDH 0.5H
43kD
34kD
HL-60Res
DMSO
M(L)+R
LY
LY+M(L)+RA
p-RAF S259
95kD
72kD
55kD
HL-60Res
DMSO
M(L)+R
LY
LY+M(L)+RA
RAF
95kD
72kD
55kD

5E

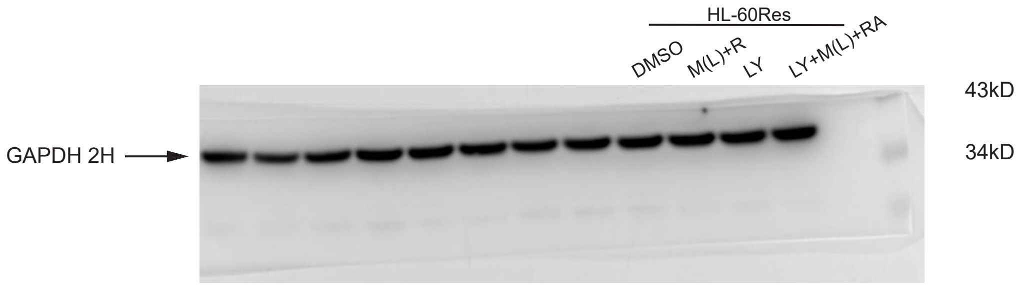

Supplemental Figure 3.

Most membranes were cut prior to hybridization. Original blots of the immunoblot detection shown in Fig 2A-Fig 2B, Fig 3D, Fig 4A-Fig 4C, Fig 5A and Fig 5E.
